# A method for partitioning trends in genetic mean and variance to understand breeding practices

**DOI:** 10.1186/s12711-023-00804-3

**Published:** 2023-06-02

**Authors:** Thiago P. Oliveira, Jana Obšteter, Ivan Pocrnic, Nicolas Heslot, Gregor Gorjanc

**Affiliations:** 1 The Roslin Institute and Royal (Dick) School of Veterinary Studies, University of Edinburgh, Edinburgh, UK; 2 Agricultural Institute of Slovenia, Ljubljana, Slovenia; 3 Limagrain, Saint-Beauzire, France

## Abstract

**Background:**

In breeding programmes, the observed genetic change is a sum of the contributions of different selection paths represented by groups of individuals. Quantifying these sources of genetic change is essential for identifying the key breeding actions and optimizing breeding programmes. However, it is difficult to disentangle the contribution of individual paths due to the inherent complexity of breeding programmes. Here we extend the previously developed method for partitioning genetic mean by paths of selection to work both with the mean and variance of breeding values.

**Methods:**

First, we extended the partitioning method to quantify the contribution of different paths to genetic variance assuming that the breeding values are known. Second, we combined the partitioning method with the Markov Chain Monte Carlo approach to draw samples from the posterior distribution of breeding values and use these samples for computing the point and interval estimates of partitions for the genetic mean and variance. We implemented the method in the R package AlphaPart. We demonstrated the method with a simulated cattle breeding programme.

**Results:**

We show how to quantify the contribution of different groups of individuals to genetic mean and variance and that the contributions of different selection paths to genetic variance are not necessarily independent. Finally, we observed that the partitioning method under the pedigree-based model has some limitations, which suggests the need for a genomic extension.

**Conclusions:**

We presented a partitioning method to quantify sources of change in genetic mean and variance in breeding programmes. The method can help breeders and researchers understand the dynamics in genetic mean and variance in a breeding programme. The developed method for partitioning genetic mean and variance is a powerful method for understanding how different selection paths interact within a breeding programme and how they can be optimised.

**Supplementary Information:**

The online version contains supplementary material available at 10.1186/s12711-023-00804-3.

## Background

Analysing genetic trends is essential for identifying key breeding actions and optimising breeding programmes. The observed genetic change is a sum of contributions from different selection paths represented by groups of individuals. However, these contributions are difficult to quantify due to the inherent complexity of breeding programmes. Contributions of selection paths differ because of differences in selection intensity, accuracy, genetic variation, generation interval, and dissemination. To quantify the contributions, García-Cortés et al. [1] developed a method for analysing the change in the genetic mean by partitioning the breeding values into the contributions of several paths. The method uses the standard partitioning of an individual’s breeding value $a_k$ into parent breeding values $\frac{1}{2}a_{f\left( k\right) }$ and $\frac{1}{2}a_{m\left( k\right) }$ and a Mendelian sampling term $w_k$.

Furthermore, the method assigns parent breeding values and Mendelian sampling terms to analyst-defined paths, such as sex, origin, selection path, etc. By aggregating these partitions by other variables, such as year, the method summarises the contributions of different groups of individuals to the overall genetic trend. This approach has been used to quantify the contributions of different countries to the overall genetic trend in the global Brown Swiss population [2], global and local Holstein populations [2], and Croatian Simmental cattle [3], Croatian Landrace, and Large-White pigs [4]. More recently, the method was used to estimate the starting point of adopting genomic selection by quantifying differences in genetic trends estimated with pedigree-based and single-step genomic best linear unbiased prediction (BLUP) [5].

In addition to the contribution of paths to changes in genetic mean, breeding programmes should also consider analysing changes in genetic variance to fully understand the source of genetic change in a population [6, 7]. Furthermore, managing the change in genetic mean and variance in breeding programmes is essential to ensure a long-term genetic gain [8, 9]. Therefore, we must quantify the contribution of different selection paths in a breeding programme to the genetic mean and variance. For example, in several economically important species, male selection and dissemination represent a crucial lever that has the largest impact on a population’s genetic mean and variance.

The aim of this paper is to extend the method of García-Cortés et al. [1] to (i) partition the genetic mean and variance, (ii) implement the method in AlphaPart R package, and (iii) apply the partitioning method to estimated breeding values following the work of [6] and [7]. We used simulation to demonstrate the methodology and provide insights on how to use the AlphaPart R package [10] to analyse real data.

## Methods

### Partitioning theory

In this section, we delve into the theory of partitioning breeding values and the computation of their mean and variance.

Let ${\mathbf{a}}$ be a vector of breeding values following a normal distribution with mean ${\mathbf{0}}$ and pedigree-based covariance ${\mathbf{A}}\sigma ^2_a$. Then, we can write ${\mathbf{a}}$ as a linear combination of the individual’s ancestor breeding values and the individual’s deviation from the ancestors ${\mathbf{a}} = {\mathbf{T}}{\mathbf{w}},$ where ${\mathbf{T}}$ is a lower-triangular matrix of expected gene flow between ancestors and individuals following a pedigree, and ${\mathbf{w}} \sim N\left( {\mathbf{0}}, {\mathbf{W}}\sigma ^2_a\right)$ are Mendelian sampling terms representing the deviations, with ${\mathbf{W}}$ being a diagonal matrix of variance coefficients and $\sigma ^2_a$ the base population (additive) genetic variance [11–14].

Assuming a factor with *p* levels, representing our paths of interest, and for any set $\sum ^{p}_{j=1}{\mathbf{P}}_{j} = {\mathbf{I}}$, García-Cortés et al. [1] partitioned the gene flow matrix into contributions of each path by defining ${\mathbf{T}}_{j}={\mathbf{T}}{\mathbf{P}}_{j}$, $j= 1, 2, \ldots , p$, and further partitioned the contribution of each path to breeding values *a priori* using the equality:

1 $${\mathbf{a}} = \left( {\mathbf{T}}_{1} + {\mathbf{T}}_{2} + \cdots + {\mathbf{T}}_{p}\right) {\mathbf{w}} = {\mathbf{a}}_1 + {\mathbf{a}}_2 + \cdots + {\mathbf{a}}_p.$$

García-Cortés et al. [1] further showed that these contributions can be estimated from data collected in breeding programmes (*a posteriori*). They first calculated the conditional expectation of breeding values given phenotype data (${\mathbf{y}}$), $\text{ EBV }=\widehat{{\mathbf{a}}}=E\left( {\mathbf{a}}|{\mathbf{y}}\right)$. Then they plugged the estimated breeding value (EBV), represented by $\widehat{{\mathbf{a}}}$, and estimated Mendelian sampling terms $(\widehat{{\mathbf{w}}})$ into Eq. (1). This approach enabled them to estimate the conditional expectation of partitions, i.e., $\widehat{{\mathbf{a}}}_j=E\left( {\mathbf{a}}_j|{\mathbf{y}}\right)$:

2 $$\widehat{{\mathbf{a}}} = \left( {\mathbf{T}}_{1} + {\mathbf{T}}_{2} + \cdots + {\mathbf{T}}_{p}\right) \widehat{{\mathbf{w}}} = \widehat{{\mathbf{a}}}_1 + \widehat{{\mathbf{a}}}_2 + \cdots + \widehat{{\mathbf{a}}}_p.$$

By summarising the breeding value partitions over time, García-Cortés et al. [1] quantified the contribution of each path (for example, males vs females, different countries, etc.) to genetic mean over time: $\mu _{a_t}$ with $t=1,2,\ldots , m$. Technically this is achieved by sub-setting the $\widehat{{\mathbf{a}}}_1, \widehat{{\mathbf{a}}}_2, \ldots , \widehat{{\mathbf{a}}}_p$ and averaging each subset to obtain ${\widehat{\mu }}_{a_{j_t}}$ where $\sum ^{p}_{j=1}{\widehat{\mu }}_{a_{j_t}} = {\widehat{\mu }}_{a_t}$.

This method has been implemented in the AlphaPart R package [10, 15]. The AlphaPart R package efficiently calculates the partitions by leveraging the sparse ${\mathbf{T}}^{-1}$ [11–14], and enables a straightforward summary by one variable, such as year, or combination of variables (interaction), such as year and sex. We refer to this variable as $x_t^{*}$, with $t=1,2,\ldots , m$, and *m* representing the number of distinct categories. Importantly, AlphaPart enables the use of any function to summarise the partitions of breeding values, i.e., $f\left( {\mathbf{a}}_j\right)$.

To enable the use of variance as one of the summary functions in AlphaPart, we extend the partitioning method to analyse the contribution of paths to genetic variance. Variance of breeding values is, *a priori*, $Var\left( {\mathbf{a}}\right) =Var\left( \mathbf{Tw}\right) =\mathbf{TWT}^{\top }\sigma ^2_a$. Using Eq. (1), we can further partition the genetic variance by paths as:

3 $$\begin{aligned} Var\left( {\mathbf{a}}\right) & = Var\left[ \left( {\mathbf{T}}_{1} + {\mathbf{T}}_{2} + \cdots + {\mathbf{T}}_{p}\right) {\mathbf{w}}\right] ,\\ & = \sum _{j=1}^{p}Var\left( {\mathbf{T}}_{j}{\mathbf{w}}\right) + 2 \sum _{j=1}^{p-1}\sum _{j^{\prime}= j+1}^{p}Cov\left( {\mathbf{T}}_{j}{\mathbf{w}}, {\mathbf{T}}_{j^{\prime}}{\mathbf{w}}\right) , \\& = \sum _{j=1}^{p}{\mathbf{T}}_{j}{\mathbf{W}}{\mathbf{T}}^{\top }_{j}\sigma ^2_{a} + 2 \sum _{j=1}^{p-1}\sum _{j^{\prime} = j+1}^{p} {\mathbf{T}}_{j}{\mathbf{W}}{\mathbf{T}}^{\top }_{j^{\prime}}\sigma ^2_{a}, \\ & = \left( \sum _{j=1}^{p} {\mathbf{a}}_{j} + 2\sum _{j=1}^{p-1}\sum _{j^{\prime}= j+1}^{p} {\mathbf{a}}_{j,j^{\prime}}\right) \sigma ^2_{a}, \end{aligned}$$

where ${\mathbf{A}}_{j} = {\mathbf{T}}_{j}{\mathbf{W}}{\mathbf{T}}^{\top }_{j}$ and ${\mathbf{A}}_{j,j^{\prime}} = {\mathbf{T}}_{j}{\mathbf{W}}{\mathbf{T}}^{\top }_{j^{\prime}}$. Note that ${\mathbf{A}}_j$ and ${\mathbf{A}}_{jj^{\prime}}$ are different from the regular numerator relationship matrix ${\mathbf{A}}$; for example, some diagonals in ${\mathbf{A}}_{j}$ and ${\mathbf{A}}_{j,j^{\prime}}$ have zero values. Note also that this partitioning of the genetic variance is similar to the multi-breed partitioning of the genetic variance [16]—we parameterise the model with one base population genetic variance. In contrast, García-Cortês and Toro [16] parameterised the model with multiple base population genetic variances and covariances.

While this partitioning by paths may involve dense matrices such as ${\mathbf{A}}_{j}$ and ${\mathbf{A}}_{j,j^{\prime}}$, we can efficiently calculate the partitions ${\mathbf{a}}_{1}, {\mathbf{a}}_{2}, \ldots , {\mathbf{a}}_{p}$ by working with the sparse ${\mathbf{T}}^{-1}$ [11–14, 17]. Again variable $x_t^{*}$ with *m* distinct categories, $t=1,2,\ldots , m$, is used to summarise the paths. Thus, we can define the genetic variance for the partition *j* given category *t* that has $n_{k} \le nI$ individuals, $k^{*}=1, 2, \ldots , n_{k}$, as:

4 $$\begin{aligned} Var\left( {\mathbf{a}}_{j_t}\right)& = E\left( {\mathbf{a}}_{j_t}^2\right) - E^2\left( {\mathbf{a}}_{j_t}\right) , \\& = \frac{1}{n_{k}}\sum _{k^{*}=1}^{n_k}\left( a_{{j_t},k^{*}} - \mu _{a_{j_t}}\right) ^2, \\& = \sigma ^2_{a_{j_t}}, \end{aligned}$$

where ${\mathbf{a}}_{j_t}$ is a column for partition *j*, but only considering individuals in category *t*, $n_k$ is the number of individuals in category *t*, and $\mu _{a_{j_t}} = \frac{1}{n_k}\sum _{k^{*}=1}^{n_k}a_{j_{t},k^{*}}$. Similarly, the genetic covariance between the partitions *j* and $j^{\prime}$, $j\ne j^{\prime}$, given category *t* is then:

5 $$\begin{aligned} Cov\left( {\mathbf{a}}_{j_t}, {\mathbf{a}}_{{j^{\prime}}_t}\right)& = E\left( {\mathbf{a}}_{j_t}{\mathbf{a}}_{{j^{\prime}}_{t}}\right) - E\left( {\mathbf{a}}_{j_t}\right) E\left( {\mathbf{a}}_{{j^{\prime}}_{t}}\right) , \\& = \frac{1}{n_k}\sum _{k^{*}=1}^{n_k}\left( a_{{j_t},k^{*}}-\mu _{a_{j_t}}\right) \left( a_{{{j^{\prime}}_{t}},k^{*}}-\mu _{a_{{j^{\prime}}_t}}\right) , \\& = \sigma _{a_{j_t}, a_{{j^{\prime}}_t}}. \end{aligned}$$

Note that the formulation of variance Eq. (4) and covariance Eq. (5) are similar to the definition in [6] but applied to breeding value partitions. By sub-setting the partitions ${\mathbf{a}}_{1}, {\mathbf{a}}_{2}, \ldots , {\mathbf{a}}_{p}$ by the variable $x^{*}_{t}$, such as year, we can calculate Eqs. (4) and (5) for each category.

It is worth noting that there is a difference between Eq. (3) and Eq. (4) or (5). The $\sigma ^2_{a}$ in Eq. (3) represents the base population genetic variance, while the expression $\left( \sum _{j=1}^{p} {\mathbf{A}}_{j} + 2\sum _{j=1}^{p-1}\sum _{j^{\prime}= j+1}^{p} {\mathbf{A}}_{j,j^{\prime}}\right) \sigma ^2_{a}$ describes how the variance changes through a given pedigree and how it partitions by paths. Equations (4) and (5) represent the variance and covariance of breeding value partitions ${\mathbf{a}}_{1}, {\mathbf{a}}_{2}, \ldots , {\mathbf{a}}_{p},$ that contribute to the total genetic variance but calculated just for individuals in the category *t*. Therefore, we can partition a population’s genetic variance into path contributions, which can be summarised in the same ways as genetic mean [1, 18]. Such analyses can quantify the contribution of different paths to changes in genetic mean and variance over time, $\mu _{a_t}$ and $\sigma ^2_{a_t}$. For example, to quantify how selection paths by sexes contribute to changes in genetic mean and variance in a breeding programme, as shown in the “Results” section, or to quantify the contribution of different countries (when importing), artificial insemination centres, or breeders.

The presented partitioning of genetic variance holds for true breeding values. However, when EBV are available, we cannot substitute ${\mathbf{a}}_1, {\mathbf{a}}_2, \ldots , {\mathbf{a}}_p$ with their expectations $\widehat{{\mathbf{a}}}_1, \widehat{{\mathbf{a}}}_2, \ldots , \widehat{{\mathbf{a}}}_p$ in Eqs. (4) and (5) as García-Cortés et al. [1] could do it for the partitioning of the genetic mean. To see this, imagine a situation where EBV are based on very limited phenotype information. Such EBV will be shrunken strongly towards zero and will have a low accuracy [14]. As such, these EBV will not be a good representation of true breeding values, and their variance, $Var(\text{ EBV})=Var\left( E\left( {\mathbf{a}}|{\mathbf{y}}\right) \right)$ will be much smaller than the variance of breeding values $\sigma ^2_{a}$ and its time trajectory $\sigma ^2_{a_t}$. To address this issue, we use the approach from Sorensen et al. [6], and Lara et al. [7] that involves three steps. First, sample breeding values from their posterior distribution [17]. Second, for every sample of breeding values, calculate desired quantities. In our case, the desired quantities are mean and variance of breeding values over time: $\mu _{a_t}$ and $\sigma ^2_{a_t}$; breeding value partitions: ${\mathbf{a}}_1, {\mathbf{a}}_2, \ldots {\mathbf{a}}_p$; and mean, variance, and covariance of the partitions over time: $\mu _{a_{j_t}}$, $\sigma ^2_{a_{j_t}}$, and $\sigma _{a_{j_t}, a_{{j^{\prime}}_t}}$. Multiple samples of these quantities represent their posterior distributions: $p\left( \mu _{a_t} | {\mathbf{y}}\right)$, $p\left( \sigma ^2_{a_t} | {\mathbf{y}}\right)$, $p\left( {\mathbf{a}}_1, {\mathbf{a}}_2, \ldots {\mathbf{a}}_p | {\mathbf{y}}\right)$, $p\left( \mu _{a_{j_t}} | {\mathbf{y}}\right)$, $p\left( \sigma ^2_{a_{j_t}} | {\mathbf{y}}\right)$, and $p\left( \sigma _{a_{j_t}, a_{{j^{\prime}}_t}} | {\mathbf{y}}\right)$. Third, summarise the samples to describe the posterior distributions of interest.

### Statistical model and computational approaches

In the previous subsection, we assumed that the true breeding values were known. However, in reality, we infer breeding values from phenotype data. To this end, we fitted the standard pedigree-based model to data described in the “Simulation” section:

6 $$\begin{aligned} {\mathbf{y}}|{\mathbf{b}},{\mathbf{a}}&\sim N\left( {\mathbf{X}}{\mathbf{b}} + {\mathbf{Z}}{\mathbf{a}}, {\mathbf{I}}\sigma ^2_{e}\right) , \nonumber \\ {\mathbf{a}}&\sim N\left( {\mathbf{0}}, {\mathbf{A}}\sigma ^2_a\right) , \end{aligned}$$

where ${\mathbf{y}}$ is a vector of observed phenotypes, ${\mathbf{b}}$ is a vector of fixed effects with the design matrix ${\mathbf{X}}$, ${\mathbf{a}}$ is a vector of breeding values with the design matrix ${\mathbf{Z}}$, $\sigma ^2_{e}$ is a residual variance, ${\mathbf{A}}$ is pedigree-based relationship matrix and $\sigma ^2_a$ is genetic variance in the base population. Additional file 1: Figs. S1 and S2 provide more information about the model definition.

We sampled from the posterior distribution of all model parameters with the Gibbs algorithm (a Markov Chain Monte Carlo (MCMC) method) as implemented in [19]. First, we constructed one chain with 80,000 samples, of which 20,000 were considered burn-in, while the remaining 60,000 were stored and thinned by saving every 40-th sample. Then, we assessed the burn-in convergence by inspecting the trace and auto-correlation plots. Consequently, 1500 samples of breeding values were stored, representing the posterior distribution $p\left( {\mathbf{a}}|{\mathbf{y}}\right)$. These samples were passed as input to the AlphaPart R package.

It is imperative to note that the proposed partitioning method requires samples from the posterior distribution $p\left( {\mathbf{a}}|{\mathbf{y}}\right)$ to enable inference of the path contributions to both genetic mean and variance. While we have used the full Bayesian approach with MCMC [17], an alternative is to use the empirical Bayesian approach; estimating variance components with restricted maximum likelihood (REML) and sampling breeding values assuming that variance components are known [7, 17]. The full Bayesian approach is recommended to account for uncertainty in estimating all model parameters.

### Frequentist measures of model fit and agreement

The partitioning methodology depends on well-calibrated estimates of breeding values. If the used model (6) does not adequately describe the data, estimates of ${\mathbf{a}}$ and derived quantities might be miss-calibrated [20]. Working with simulation, we have the benefit of knowing the true breeding value of individuals (${\mathbf{a}}$) and can hence evaluate how our estimates of breeding values are calibrated.

First, we evaluated the agreement between true and estimated mean and variance of breeding values over generations using the concordance correlation coefficient defined by [21]. Let *t* be the index for the generation with $t=1, 2, \ldots , m$. Recall the mean and variance of true breeding values at generation *t* respectively as $\mu _{a_t}$ and $\sigma ^2_{a_t}$. Moreover, let $\widehat{{\mathbf{a}}} = E\left( {\mathbf{a}}|{\mathbf{y}}\right)$ be the vector of posterior means of individual breeding values in $p\left( {\mathbf{a}}|{\mathbf{y}}\right)$, and $E\left( \widehat{{\mathbf{a}}}_{t}\right)$ and $Var\left( \widehat{{\mathbf{a}}}_{t}\right)$, respectively, be the mean and variance of these posterior means at generation *t*. We then evaluated the agreement between the variables ${\mathbf{Y}}^{*}_{1_t} = \left( \mu _{a_1}, \mu _{a_2}, \ldots , \mu _{a_m}\right) ^{\top }$ and ${\mathbf{Y}}^{*}_{2_t} = \left( E\left( \widehat{{\mathbf{a}}}_{1_t}\right) , E\left( \widehat{{\mathbf{a}}}_{2_t}\right) , \ldots , E\left( \widehat{{\mathbf{a}}}_{m_t}\right) \right) ^{\top }$ and between the variables ${\mathbf{Y}}^{*}_{1_t} = \left( \sigma ^2_{a_1}, \sigma ^2_{a_2}, \ldots , \sigma ^2_{a_m}\right) ^{\top }$ and ${\mathbf{Y}}^{*}_{2_t} = \left( Var\left( \widehat{{\mathbf{a}}}_{1_t}\right) , Var\left( \widehat{{\mathbf{a}}}_{2_t}\right) , \ldots , Var\left( \widehat{{\mathbf{a}}}_{m_t}\right) \right) ^{\top }$. Assuming that the pairs of $\left( Y_{1_t}^{*}, Y_{2_t}^{*}\right)$ are independent draws from a bi-variate population with means $\mu _1$ and $\mu _2$ and a covariance matrix:

$$Cov(Y_{1_t}^{*}, Y_{2_t}^{*}) =\left( \begin{array}{cc} \sigma _{1}^2 &{} \sigma _{12} \\ \sigma _{12} &{} \sigma _{2}^2 \end{array} \right) ,$$

we can evaluate the agreement between ${\mathbf{Y}}_{1_t}^{*}$ and ${\mathbf{Y}}_{2_t}^{*}$ with the concordance correlation coefficient [21]. This coefficient lies between $-1$ and 1, and is given by:

$$\begin{aligned} \rho _{c} =\frac{2\sigma _{12}}{\sigma _{1}^{2}+\sigma _{2}^{2}+\left( \mu _{1}-\mu _{2}\right) ^2} \end{aligned}$$

where $\mu _{1}=\text{ E }\left( {\mathbf{Y}}^{*}_{1_t}\right)$, $\mu _{2}=\text{ E }\left( {\mathbf{Y}}^{*}_{2_t}\right)$, $\sigma _{1}^{2}= \text{ Var }\left( {\mathbf{Y}}^{*}_{1_t}\right)$, $\sigma _{2}^{2}= \text{ Var }\left( {\mathbf{Y}}^{*}_{2_t}\right)$, and $\sigma _{12}=\text{ Cov }\left( {\mathbf{Y}}^{*}_{1_t}, {\mathbf{Y}}^{*}_{2_t}\right)$. It can be shown that $\rho _c= \rho \times C_b$, where $\rho$ is the Pearson correlation coefficient, and $C_b$ is the bias correction factor. Here, $\rho$ measures how far each observation deviates from the best-fit line, and $C_b \in [0,1]$ measures how far the best-fit line deviates from the identity line $y=x$ and is defined as $C_b=2\left( v+v^{-1}+u^{2}\right) ^{-1}$, where $v = \sigma ^2_{1}/\sigma ^2_{2}$ is a scale shift and $u = (\mu _{1} - \mu _{2}) / \sqrt{\sigma _1\sigma _2}$ is a location shift relative to the scale. When $C_b=1$, there is no deviation from the identity line, consequently, the quantity of interest is close to the ‘truth’. We also used root mean square deviation (RMSD) to measure the bias between ${\mathbf{Y}}^{*}_{2_t}$ and ${\mathbf{Y}}^{*}_{1_t}$, which is given by:

$$\text{ RMSD } = \left[ \frac{1}{m}\left( {\mathbf{Y}}^{*}_{2_t} - {\mathbf{Y}}^{*}_{1_t}\right) ^{\top }\left( {\mathbf{Y}}^{*}_{2_t} - {\mathbf{Y}}^{*}_{1_t}\right) \right] ^{1/2}.$$

We also evaluated the distribution of the difference between true and estimated quantities of interest. We show this evaluation for mean and variance of breeding value partitions over various categories (sex and generation in the example described in the following). Let $n_r$ be the number of simulation replicates, $r = 1, 2, \ldots, n_r$ and ${\mathbf{a}}_{j_t,r}$ the partition of breeding values for the path *j* category of individuals *t* in replicate *r*. We obtained the posterior distribution of our quantities of interest for the partitions and categories in each replicate: $p\left( \mu _{a_{j_t,r}}|{\mathbf{y}}_r\right)$, $p\left( \sigma ^2_{a_{j_t,r}}|{\mathbf{y}}_r\right)$, and $p\left( \sigma _{a_{j_t,r}},{a_{{j^{\prime}}_t,r}}|{\mathbf{y}}_r\right)$, summarised these posterior distributions with the posterior mean, and calculated the difference between this posterior mean and the corresponding true value, for example, $\mu _{a_{j_t,r}} - E\left( \mu _{a_{j_t,r}}|{\mathbf{y}}_r\right)$. With a good model fit, we expect that the difference is centred around zero.

### AlphaPart implementation

The partitioning method is implemented in the AlphaPart R package [10, 18]. The main input for the analysis is a data frame (data) with:

- pedigree information for individual (id), sire (Fid) and dam (Mid);
- partition variable (path)—colPath;
- breeding values for one or multiple traits—colBV;
- grouping variable ($x^{*}_{t}$) used to compute the conditional expectations such as generation, birth year, location, etc.

We partition the breeding values (BV) by paths using:
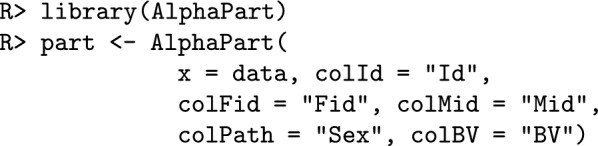


We summarise the partitions using the grouping variable (time) using:
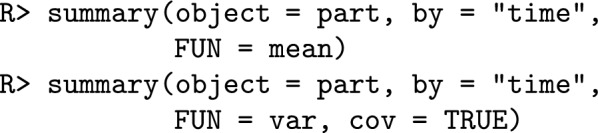


where object is an object of class AlphaPart with breeding value partitions, by represents the column by which summary function FUN is applied. For this work, we included an extra argument cov that controls how the covariances are displayed in the output. If cov = FALSE, the default, all covariances are returned in a single column as $2\sum _{j=1}^{p-1}\sum _{j^{\prime}= j+1}^{p} Cov (a_j, a_{j^{\prime}})$, otherwise, if cov = TRUE, the summary method returns $p\left( p-1\right) /2$ columns, where each column represents covariances as $2 Cov(a_j, a_{j^{\prime}} )$.

**Fig. 1:**
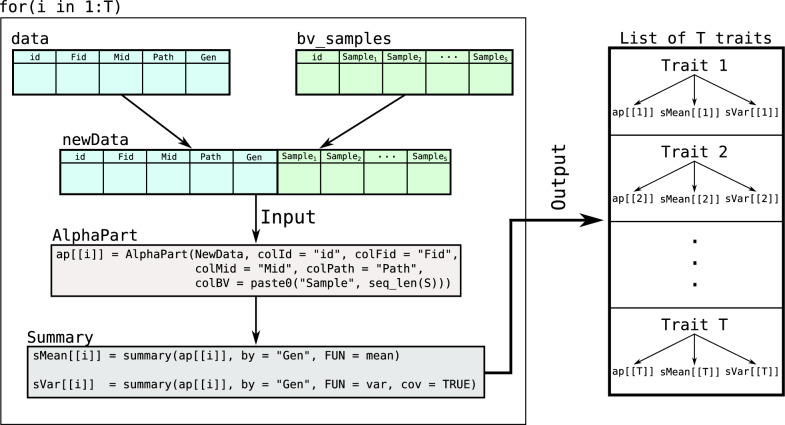
Flowchart representing a possible algorithm to evaluate contributions of paths to genetic mean and variance using samples of breeding values with the AlphaPart R package in a multi-trait case

We further describe how to use the posterior samples of breeding values from the “Statistical model and computational approaches” section in AlphaPart. Let *T* be the number of traits and *S* be the number of samples of breeding values. Suppose data is a data frame containing columns for individual (id), father (Fid), mother (Mid), path (path), and generation (Gen). Now suppose a more general case where bv_samples represents a data frame containing a column for the individual (id) identification and *S* columns for the samples of breeding values, as shown in Fig. 1. To prepare the input data for AlphaPart, we can merge the data frames called data and bv_samples into a new data frame called newData (Fig. 1). We can then use the function AlphaPart() to calculate breeding value partitions with the difference that we now should pass the names of the samples to the argument colBV (Fig. 1). Afterwards, the summary() function can be called to summarise the partitions using an explanatory variable, such as generation (Gen). Since we work with posterior samples of breeding values, we obtain posterior samples for the summaries of the partitions (see the accompanying code). Finally, in the case with more than one trait, we suggest a for loop (possibly parallelised) to create one output per trait, as shown in Fig. 1. In an extreme case with more traits than samples, an alternative approach would be to save one sample of breeding values for multiple traits in one data frame and loop over the samples.

### Simulation

**Fig. 2:**
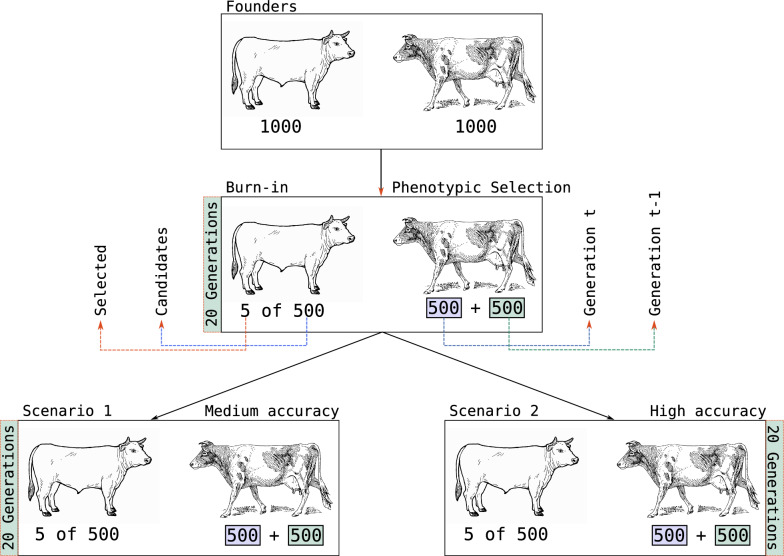
Simulation scheme illustrating an overview of the medium- and high-accuracy scenarios

To evaluate the method and AlphaPart implementation, we simulated a simple cattle breeding programme over 40 generations with 1000 individuals per generation. The first 20 years represented a burn-in phase, where we selected the best 5 males (out of 500) as sires based on their phenotype and mated them with all 500 females from the previous generation and all 500 females from the current generation. These matings produced 1000 selection candidates for the next generation. After the burn-in phase, we tested two selection scenarios over a further 20 generations: we selected the 5 best males from 500 male candidates based on (i) their phenotypes (‘medium-accuracy’ scenario, $r = 0.3$) or (ii) true breeding values (‘high-accuracy’ scenario, $r = 1$), as shown in Fig. 2. We replicated the simulation 30 times with the same founding genomes.

The simulation was done with the AlphaSimR R package version 1.0 [22]. We simulated a cattle genome from the coalescent model with recombination and Holstein demography [23]. The genome had 30 chromosomes and 30,000 quantitative trait loci (QTL). The QTL were randomly sampled from segregating sites and had an additive effect sampled from a normal distribution for a single-trait phenotype with a heritability of 0.3. The above-described breeding programme has a low effective population size (ignoring that we use females for two generations $N_e\sim ( 4\times nSires \times nDams) / (nSires + nDams) = (4 \times 5 \times 1000) / (1005) < 20$) because our aim was to generate an intense selection situation that would show changes in genetic mean and variance. We split the gene-flow matrix ${\mathbf{T}}$ by specifying male and female paths $\left( {\mathbf{P}}_{m}+{\mathbf{P}}_f = {\mathbf{I}}\right)$. Furthermore, we split the male path into selected and non-selected path $\left( {\mathbf{P}}_m^{s}+{\mathbf{P}}_{m}^{n} = {\mathbf{P}}_{m}\right)$, where ${\mathbf{P}}_{m}$ is a diagonal matrix with 1s in rows for males and zeros otherwise; ${\mathbf{P}}_{f} = {\mathbf{I}} - {\mathbf{P}}_{m}$; ${\mathbf{P}}_{m}^{s}$ is a diagonal matrix with ones in rows for selected males, and ${\mathbf{P}}^{n}_{m} = {\mathbf{P}}_m-{\mathbf{P}}_{m}^{s}$ is a diagonal matrix with 1s in rows for non-selected males. To facilitate interpretation, we scaled the genetic mean and variance of the base population, respectively, to 0 and 1.

### Software implementation

We simulated the cattle breeding programme using AlphaSimR R package [22]. We fitted the model in Eq. (6) using the BLUPF90 family of programs [19], while all post-processing was done in R [15]. To compute and summarise the partitions, we used the AlphaPart R package [10], to prepare data and present results, we used the collection of tidyverse R packages [24] and patchwork R package [25]. The simulation and analysis code is fully available at the GitHub repository https://github.com/HighlanderLab/toliveira_alphapart_variance.

## Results

### Partitioning of true breeding values

**Fig. 3:**
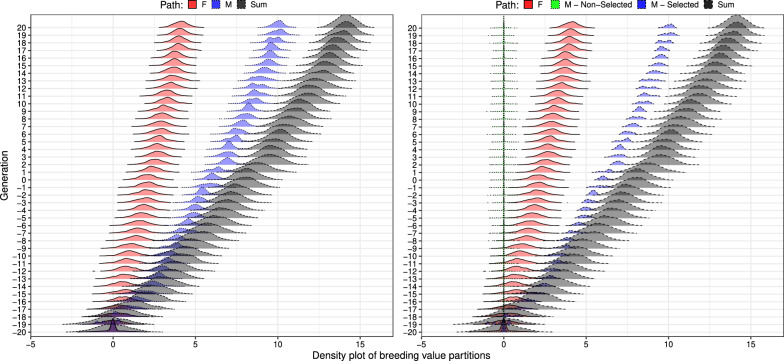
Distribution of breeding value partitions by sex and by sex and selection status [selected males (M(S)), non-selected males (M(N)), and females (F)] over generations for medium-accuracy scenario

Analysing true breeding values is essential to demonstrate how the partitioning of breeding values and their means and variances works without the uncertainty of estimating breeding values. Figure 3 shows distributions of true breeding values and partitions over generations for the medium-accuracy scenario. While we partitioned true breeding values, simulation was driven by selection with medium or high accuracy. The accuracy impacted true trends in genetic mean and variance, and we analysed these simulation outputs.

Figure 3a shows partitions for female and male paths. As expected, the male path contributed the most to genetic gain, almost twice as much as the female path. Even though there was no selection between females (all females contributed progeny for two generations), the contribution of the female path was significantly different from zero. This shows that nevertheless the female path contributed to the genetic gain, as we will analyse further below.

**Fig. 4:**
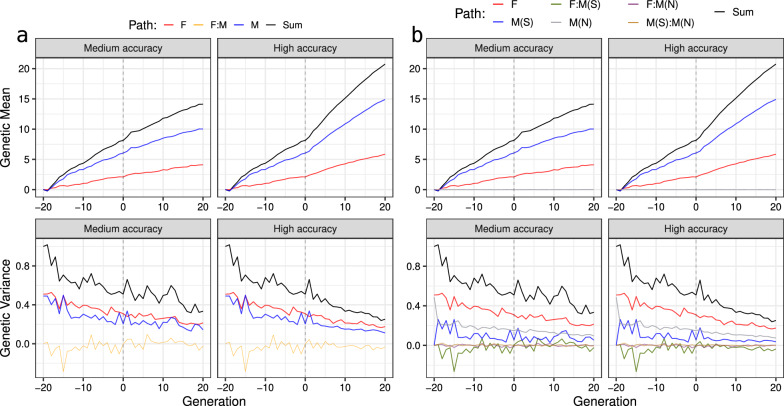
Partitions of genetic mean and variance by **a** sex (males and females) and **b** by sex and selection status [selected males (M(S)), non-selected males (M(N)), and females (F)] using true breeding values for one simulation replicate

We now turn attention to the summary of the partitions from Fig. 3 with a mean and variance shown in Fig. 4, focusing on the medium-accuracy scenario. Means of partitions followed the centre of distributions shown in Fig. 3. In contrast, partitioning variances indicated a smaller variation for the male path than for the female path, in line with only male selection in our example. However, trends of partitioned variances in Fig. 4a suggest that the variance of both male and female paths are very similar. This observation raises a question: “How can male and female paths contribute similarly to the genetic variance over time if we were selecting only between males?”. The answer to this question is shown in Figs. 3b and 4b, where we partitioned breeding values by sex and selection status. Clearly, non-selected males do not contribute to the change in the genetic mean because their Mendelian sampling terms are distributed around zero in their generation and do not contribute to future generations. However, non-selected males still contribute to the genetic variance in their generation, yet this variation is not passed to the next generation. To separate this temporary contribution to genetic variance, we must define path variables by sex and selection status. By doing this, we see that the main source of change in genetic variance are the five selected males, as expected (Fig. 4b).

The higher accuracy scenario expectedly drove more significant changes in genetic mean and variance than the medium accuracy scenario (Fig. 4). This comparison shows that the contribution of paths to genetic variance is a function of selection accuracy, with higher accuracy driving more changes in genetic variance. Notably, with medium accuracy, we saw a smaller difference between partitions of genetic variance for selected and non-selected males. The main reason for this is that the medium accuracy likely did not enable the selection of the top males from the tail of the distribution, which would have had a much smaller variance. We show the full distribution of the partitioned breeding values in Additional file 2: Fig. S3 and Additional file 3: Fig. S4 over 40 generations.

Splitting the male path into selected and non-selected paths also showed that the negative covariance between male and female partitions in Fig. 4a was driven by the covariance between female and selected male partitions (F:M(S), Fig. 4b). This covariance was consistently negative from generation 8 to 20 in the high-accuracy scenario (Fig. 4b), resulting in a mean correlation of $-0.33$ ($\pm 0.15$) for those generations. As a result, the total genetic variance in a generation *t* can be smaller than the sum of genetic variances for partitions. This non-independence of partitions of genetic variance is more evident in the high-accuracy scenario from generations 8 to 20, where the correlation decreased even more than in the medium-accuracy scenario (see Additional file 4: Fig. S5). The non-independence of partitions of genetic variance is yet another reason why individual partitions of genetic variance must be interpreted with caution. We return to this point in the discussion.

**Fig. 5:**
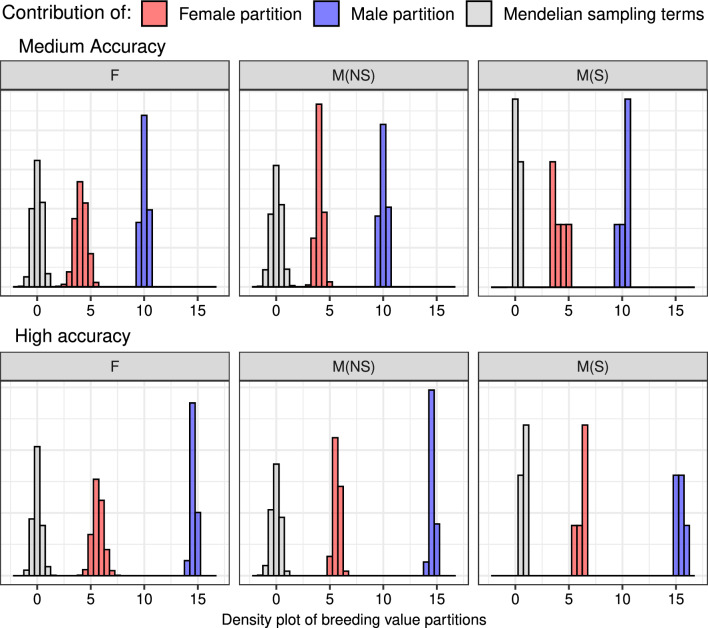
Distribution of breeding value partitions and Mendelian sampling terms by path in generation 39 for medium-accuracy (**a**) and high accuracy (**b**) selection scenarios

To further clarify why female partition had a non-zero contribution to the genetic gain, in spite of the absence of selection among females, Fig. 5 shows the histogram of breeding value partitions and Mendelian sampling terms by the path in generation 39 for medium-accuracy (Fig. 5a) and high-accuracy (Fig. 5b) scenarios. We can see that the female partition contributed significantly to genetic gain (red distribution), although less than the selected males’ partition (blue distribution), in each group of individuals (females, non-selected males, and selected males). Expectedly, Mendelian sampling terms for females and non-selected males were distributed around zero (gray distribution), while selected males had consistently positive Mendelian sampling terms. However, females were the progeny of previously selected males, and their sons were subject to selection, which created a non-zero contribution for the female partition (red distribution)—through the dissemination of genes selected in their sires and through their (dam’s) sons.

The presented results showed one replicate of the simulation. In Additional file 5: Fig. S6, we show the partitioning analysis for each of the 30 replicates that all used identical founding genomes. Our aim was to show that the above results are consistently observed across many replicates but also to show the magnitude of variation between replicates. The solid line represents the median, and the ribbon represents the distribution of true partitions of genetic mean and variance and the correlation between selected male and female partitions.

### Estimating the partitions of genetic mean and variance

#### Model fit

The data were analysed with model (6) using the complete pedigree that enabled accurate estimation of residual and base population genetic variance. However, we slightly overestimated base population genetic variance in the high-accuracy scenario (Table 1). Evaluating the model further in terms of estimating the quantities of interest, we observed that estimates under the medium accuracy scenario for genetic means over generations were better calibrated than for genetic variance over generations (Table 2). Under the high-accuracy scenario, the genetic mean over generations was also well estimated, but there was considerable miss-calibration for the genetic variance over generations (Table 2). The estimated and true genetic means and variances over 40 generations are shown in Additional file 6: Fig. S7 and Additional file 7: Fig. S8. One reason for a worse performance of model (6) under the high-accuracy scenario was that it generated significant genetic change both in mean and variance (Fig. 4, which was also manifested by a higher level of inbreeding than the medium-accuracy scenario (see Additional file 8: Fig. S9). As inbreeding increases over generations, it generates variation between individuals that is challenging to represent using only pedigree-based relationships and better approaches are needed, such as genomic relationships.

**Table 1:** Variance components (VC) true values, point estimates (posterior mean), and 95% highest posterior density (HPD) interval

Scenario	VC	True	Estimate	95% HPD	
				Lower	Upper
Medium accuracy	$\sigma ^2_a$	0.3	0.27	0.25	0.30
	$\sigma ^2_e$	0.7	0.69	0.68	0.71
High accuracy	$\sigma ^2_a$	0.3	0.35	0.33	0.38
	$\sigma ^2_e$	0.7	0.66	0.64	0.67

**Table 2:** Estimate and 95% confidence interval for the concordance correlation coefficient (${\widehat{\rho }}_{\tiny {\text{ c }}}$) between the true and estimated statistic, and point estimates for the Pearson correlation coefficient (${\widehat{\rho }}$); bias correction factor (${\widehat{C}}_b$); and root mean square deviation (RMSD) in each case within scenario

Scenario	Statistic	Concordance correlation			${\widehat{\rho }}$	${\widehat{C}}_{b}$	RMSD		
		${\widehat{\rho }}_{\tiny {\text{ c }}}$	Lower	Upper			Est.	Lower	Upper
Medium accuracy	$\mu _{a_t}$ vs. $E\left( \widehat{{\mathbf{a}}}_t\right)$	1.00	1.00	1.00	1.00	1.00	0.10	0.08	0.15
	$\sigma ^2_{a_t}$ vs. $Var\left( \widehat{{\mathbf{a}}}_t\right)$	0.95	0.93	0.96	0.96	0.99	0.11	0.09	0.14
High accuracy	$\mu _{a_t}$ vs. $E\left( \widehat{{\mathbf{a}}}_t\right)$	1.00	1.00	1.00	1.00	1.00	0.15	0.11	0.20
	$\sigma ^2_{a_t}$ vs. $Var\left( \widehat{{\mathbf{a}}}_t\right)$	0.87	0.83	0.90	0.97	0.89	0.18	0.15	0.21

#### Genetic means and its partitions

**Fig. 6:**
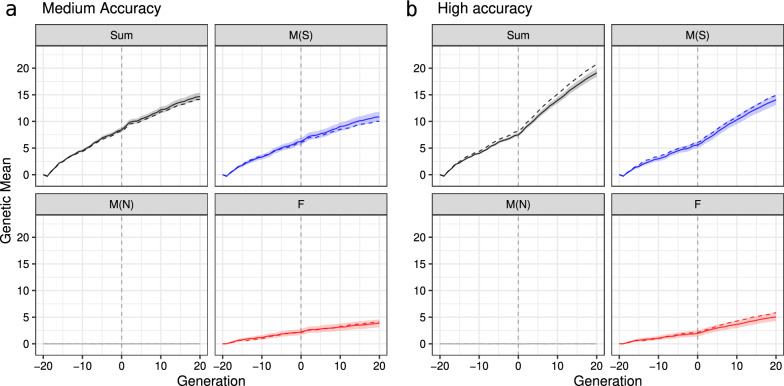
Partitioning of the total genetic mean (Sum) over generations by selected males (M(S)), non-selected males (M(N)), and females (F) paths in the medium-accuracy (**a**) and high-accuracy (**b**) selection scenario, considering one replicate (true value is denoted with a dashed line and posterior mean denoted with a solid line and 95% credible interval is denoted with a ribbon)

**Fig. 7:**
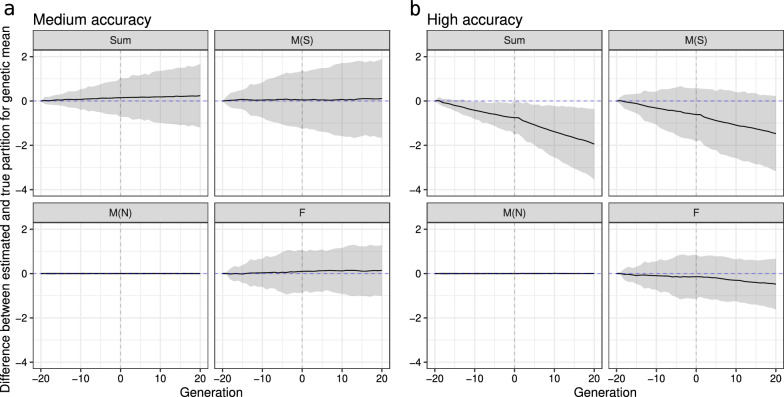
Distribution of the difference between true and estimated genetic means over generations for the total (Sum) partitioned by selected males (M(S)), non-selected males (M(N)), and females (F) paths in the medium-accuracy (**a**) and high-accuracy (**b**) selection scenario, considering 30 replicates (zero value is denoted with a dashed line and mean difference over replicates is denoted with a solid line and 95% quantile of differences over replicates is denoted with a ribbon)

Now that the adequacy of model (6) has been assessed and its impact on the estimates of genetic means and variances over generations has been evaluated, we show the partitioning results when breeding values are estimated from phenotypes. First, we illustrate partitioning results from a single replicate, then extend it by showing results from 30 replicates. Figure 6 shows the true and estimated genetic mean over 40 generations for the medium- and high-accuracy scenarios considering the total genetic mean (Sum), the path for selected males (M(S)), non-selected males (M(NS)), and females (F). For the medium-accuracy scenario, although the point estimate for the mean of selected males partition showed underestimation), the true means of partition of each path was within the 95% credible interval. For the high-accuracy scenario, we observed underestimation for females and selected males partition. Consequently, the underestimation of the total genetic mean was even higher because it is the sum of those two contributions, while non-selected males had a zero contribution. Figure 7 confirms this result by showing the difference between true and estimated means of partition over 30 replicates. Additional file 9: Fig. S10 shows that the observed deviations in both scenarios do not come from inadequately estimated Mendelian sampling terms. Hence, the source of error must be due to the inadequate estimation of the parent average terms.

#### Genetic variance and its partitions

**Fig. 8:**
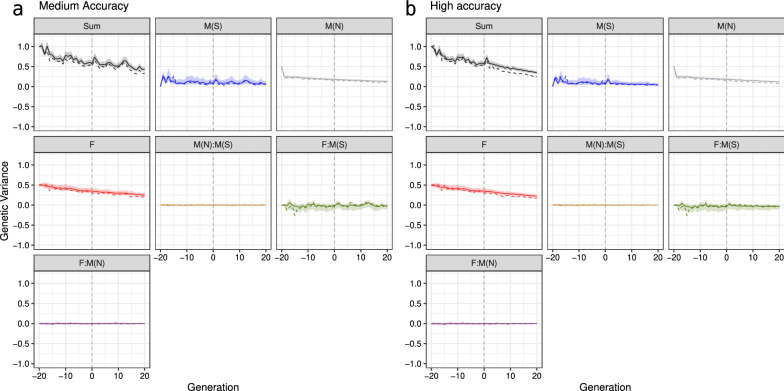
Partitioning of the total genetic variance (Sum) over a generation by selected males (M(S)), non-selected males (M(N)), and females (F) path in the medium-accuracy (**a**) and high-accuracy (**b**) selection scenario, considering one replicate (true value is denoted with a dashed line and posterior mean denoted with a solid line and 95% credible interval is denoted with a ribbon)

**Fig. 9:**
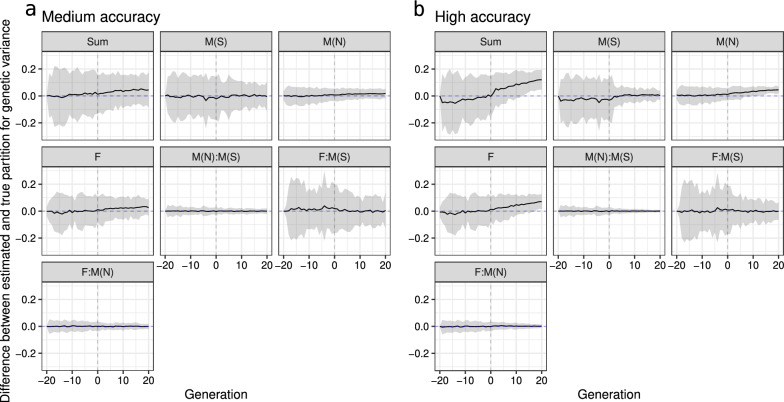
Distribution of the difference between true and estimated genetic variances over generations for the total (Sum) partitioned by selected males (M(S)), non-selected males (M(N)), and females (F) paths in the medium-accuracy (**a**) and high-accuracy (**b**) selection scenario, considering 30 replicates (zero value is denoted with a dashed line and mean difference over replicates is denoted with a solid line and 95% quantile of differences over replicates is denoted with a ribbon)

The partitioning of genetic variance by paths in the medium- and high-accuracy scenarios in a single replicate are shown in Fig. 8. While we correctly estimated the overall trends in the total genetic variance and its partitions, we observed a slight overestimation for the female’s and non-selected male’s paths and its total in either the medium- or high-accuracy scenarios. However, from generation 1 to 20 in the high-accuracy scenario, the overestimation increased compared to the medium-accuracy scenario. These observations were also confirmed across 30 replicates for both scenarios (Fig. 9). Importantly, distribution over 30 replicates did not include zero in later generations indicating significant differences in the estimates from the true values. Figure 9 also shows an underestimation of genetic variance for the selected male’s path in early generations (− 19 to 2), which leads to the underestimation of the total genetic variance in the high-accuracy scenario.

We have initially observed even larger differences but have addressed these by adequately accounting for inbreeding in setting up the ${\mathbf{A}}^{-1}$. Ignoring inbreeding significantly impacted the estimates of genetic means and variances and their partitions (see Additional file 10: Fig. S11, Additional file 11: Fig. S12, Additional file 12: Fig. S13).

## Discussion

We developed a method for partitioning the trends in genetic variance into contributions of different paths as an extension of the previous work with trends in the genetic mean of García-Cortés et al. [1] and Obsteter et al. [18]. The method used to infer the path contributions over generations is illustrated using a single-trait model; however, extension to multiple traits is straightforward and already implemented in AlphaPart. The extension presented here allows researchers to quantify the drivers of genetic variance in their breeding programmes in addition to the drivers of the genetic mean. Consequently, it could help quantify the dynamics between genetic mean and variance in global animal breeding [2, 4], how different breeding schemes impact their long-term sustainability [26, 27], and how much variability is introgressed in pre-breeding programmes [28, 29]. Therefore, it is a powerful and valuable method for retrospective analysis and understanding how different groups of breeding individuals contribute to change in genetic mean and variance, a topic that has been discussed in the last few years [5, 7, 30]. Moreover, the partitioning analysis can contribute to future decisions in breeding strategies through analysis of past real data or by analysing a combination of real and simulated data to make inferences about future results. For this reason, we implemented this method in the AlphaPart R package. The extension has been available since version 0.9.3, and is freely available from CRAN.

The simulated cattle breeding programme with the medium- and high-accuracy scenarios illustrated the power of the partitioning method to summarise genetic trends in mean and variance. However, some care is needed when using the proposed method. We have shown that the path variable must be considered carefully because a specific choice can lead to a misinterpretation of the contributions, especially regarding the partition of genetic variance. To this end, we recommend plotting the distribution of partitioned breeding values, where partitions can be done with different variables of interest, like sex and selection status, in our study.

By partitioning the genetic mean and variance, we showed that in the high-accuracy scenario, the covariance between contributions of females and selected males plays an important role when partitioning the genetic variance. Consequently, in this case $Var\left( {\mathbf{a}}\right) < Var\left( {\mathbf{a}}_F\right) + Var\left( {\mathbf{a}}_M\right)$, where *F* and *M* represent the female and male paths. Furthermore, most of the (additive) genetic variance in the breeding programme pertained to female and non-selected male paths, which were not the most relevant individuals for disseminating genetic gain, indicating that the selected male path drove changes in genetic mean. While this is an obvious result, it shows the power of the method for more complex cases. A negative correlation between female and selected male partitions in Fig. 4 and Additional file 4: Fig. S5 means that the partitions of genetic variance are not independent. Since the male partition contributes more and more over generations, the female partition has to contribute less, which induces negative covariance between them. In this sense, we demonstrated that variance partitions are not necessarily independent; therefore, they should not be analysed separately.

A negative covariance between breeding value partitions is expected in some cases. We are aware of two cases. The first is when paths represent sexes, as in this study. The second is when paths represent a foreign and a domestic breeding programme. Covariance arises from the proportional relationship between contribution of paths as well as their values, as shown in Additional file 13: Fig. S14 (case A). To illustrate this in the context of sex paths, assume we are mating the best male with a female. In this case, it is expected that the male path will contribute more to the next generation due to the higher intensity (and sometimes accuracy) of selection. Consequently, sires are often the main drivers of genetic change in a population. On the other hand, since the proportion of gene contribution from male and female paths to an offspring must sum to 1, if males contribute more to the value of the next generation, then females must contribute less. This relationship induces negative covariance. The same happens with foreign and domestic paths, assuming that we are importing individuals with high breeding values into a population. Suppose these individuals are well adapted to the environment of the population. In that case, the contribution of the foreign path will increase over time, and the contribution of the domestic path will decrease. This relationship will also induce negative covariance.

A positive covariance is not likely to happen when paths represent sexes in a target population for a reason explained in the previous paragraph. However, it can happen when we import individuals that are not well-adapted to the domestic environment. Such individuals contribute negatively to the next generation of offspring (Additional file 13: Fig. S14 case B). In this case, the best genetic material from both domestic and imported paths is expected to contribute more to the next generation, which generates a positive covariance between the two paths that move in tandem in the same direction. Therefore, a positive covariance could be used to alert breeders about the negative impact of introgression since some imported animals are harming domestic genetic gain.

The results showed the overestimation of estimated partitions for genetic variance in the high-accuracy scenario, which originated from the model’s lack of fit to the data as quantified by the too-high estimate of the base population genetic variance (Table 1) and low concordance correlation coefficient for estimates of genetic variance over generations (Table 2). While our example is extreme with a low effective population size, it shows the importance of accurately estimating model parameters in populations under selection [31, 32]. Namely, the quality of model parameters estimates impacts the downstream analyses, such as the partitioning of breeding values in this study.

This overestimation of the base population genetic variance and its partitions in the high-accuracy scenario is likely impacted by the lack of information in the pedigree-based model for such an intense selection and low effective population size ($\hbox {Ne} < 20$) simulated in our study [12]. Namely, we have observed significant changes in the genetic variance of up to 75% over 40 generations. While the pedigree-based model can account for selection [31, 32], it does not seem to account appropriately for such a significant change in genetic variance [6, 7, 12]. Therefore, our next step is to develop an extension of the partitioning method considering genomic data to overcome the issue of working with the expected probability of identity by descent from pedigrees by using the realised identity by descent or state from genomic data [33, 34]. We have recently already extended the Sorensen et al. [6] method for temporal estimation of genetic variance with a pedigree-based model to work with genomic data. This extension enables quantifying changes in genetic variance due to changes in allele frequencies caused by drift and selection and changes in linkage-disequilibrium caused by selection (the Bulmer effect). Extending the partitioning method of García-Cortés et al. [1] and current work with such genomic insights is a natural next step.

## Conclusions

We developed a method to quantify the drivers of genetic variance in breeding programmes by partitioning the genetic variance by analyst-defined paths. The method developed can provide a comprehensive overview of breeding practises, either based on past results or through simulated scenarios, as shown in this study. Moreover, the covariance between paths can inform the breeder about the dynamics of contributions and can be used to identify potential pitfalls of the breeding programme.

The method can be easily applied to real data by leveraging established software to draw posterior breeding values samples given the observed phenotype data. Working with the posterior sample of breeding values also enables straightforward uncertainty quantification in evaluated partitions and their summaries, mean and variance.

We observed some overestimation of genetic variance and its partitions, but this was caused by the extreme selection in our simulation study and the pedigree-based model, which showed a lack of fit with respect to the observed genetic change in mean and variance. Our future research will extend the proposed method using genomic data to overcome the limitations of the pedigree-based model under such extreme selection settings.

## Supplementary Information

**Additional file 1: Figure S1.** Definition of the statistical model, priors and posteriors: Directed acyclic graph of the pedigree-based model with *nI* individuals and *nY* phenotypic records;" title="Click here to edit">) with explicit representation of Mendelian sampling terms;" title="Click here to edit">) and error term;" title="Click here to edit">), where $\sigma ^2_a$ is the additive genetic variance, $a_{f}$ and $a_{m}$ are the parent’s breeding value, **1** represents a vector of ones, $\mu _i$ the linear predictor, and $\sigma ^2_e$ the residual variance. **Figure S2.** Definition of the statistical model, priors and posteriors: representation of gender as the path variable.

**Additional file 2: Figure S3.** Distribution of breeding value partitions by sex and selection status [selected males), non-selected males), and females] over generations for medium-accuracy scenario [35].

**Additional file 3: Figure S4.** Distribution of breeding value partitions by sex and selection status [selected males), non-selected males), and females] over generations for high-accuracy scenario.

**Additional file 4: Figure S5.** Correlation between females (F) and selected males (M(S)) partitions using true breeding values for the medium- and high-accuracy scenarios and one simulation replicate.

**Additional file 5: Figure S6.** Partitions of genetic mean and variance bysex,by sex and selection status [selected males), non-selected males), and females], andthe Pearson correlation between F and Mpartitions for the medium- and high-accuracy scenarios by sex and selection status using true breeding values for 30 simulation replicates.

**Additional file 6: Figure S7.** Estimated and true genetic means and variances over 40 generations by selected males), non-selected males), and femalesin the medium-accuracy scenario. The solid line represents the equality line $y=x$, and the dots are the Cartesian coordinates of estimated and true values.

**Additional file 7: Figure S8.** Estimated and true genetic means and variances over 40 generations by selected males), non-selected males), and femalesin the high-accuracy scenario. The solid line represents the equality line $y=x$, and the dots are the Cartesian coordinates of estimated and true values.

**Additional file 8: Figure S9.** Pointand intervalestimates for inbreeding over generation considering all animals in a specific generation.

**Additional file 9: Figure S10.** The difference between true and estimated Mendelian sampling termsis distributed over generations. The totalis partitioned by selected males), non-selected males), and femalespaths in the medium- and high-accuracy selection scenario. We are considering 30 replicates (zero value is denoted with a dashed line and mean differenceover replicates is denoted with a solid line, and 95% quantile of differences over replicates is denoted with a ribbon).

**Additional file 10: Figure S11** Partitioning of the total genetic meanover generations by selected males), non-selected males), and femalespaths in the medium-accuracy and high-accuracy scenario. We considered one replicate without accounting for inbreeding in the model (true value is denoted with a dashed line and posterior mean denoted with a solid line, and 95%credible interval is denoted with a ribbon).

**Additional file 11: Figure S12.** Partitioning of the total Mendelian Sampling termover generations by selected males), non-selected males), and femalespaths in the medium-accuracy and high-accuracy scenario. We considered one replicate without accounting for inbreeding in the model (true value is denoted with a dashed line and posterior mean denoted with a solid line,and 95% credible interval is denoted with a ribbon).

**Additional file 12: Figure S13.** Partitioning of the total genetic varianceover a generation by selected males), non-selected males), and femalespath in the medium-accuracy and high-accuracy scenario. We considered one replicate without accounting for inbreeding in the model (true value is denoted with a dashed line and posterior mean denoted with a solid line, and 95%credible interval is denoted with a ribbon).

**Additional file 13: Figure S14.** Example ofnegative andpositive covariance partitions.

## Data Availability

Project name: AlphaPart; Project home page: https://cran.r-project.org/package=AlphaPart; Operating system(s): Windows, MacOS, Linux; Programming language: R & C++; Licence: GPL-3; Data and Code: https://github.com/HighlanderLab/toliveira_alphapart_variance.

## References

[1] García-Cortés LA, Martínez-Ávila JC, Toro MA (2008). Partition of the genetic trend to validate multiple selection decisions. Animal.

[2] Gorjanc G, Potocnik K, García-Cortés LA, Jakobsen J, Dürr J (2011). Partitioning of international genetic trends by origin in brown swiss bulls. Interbull Bull..

[3] Špehar M, Ivkic Z, Bulic V, Barac Z, Gorjanc G (2011). Partitioning of genetic trends by origin in Croatian Simmental cattle. Agric Conspec Sci.

[4] Škorput D, Gorjanc G, Kasap A, Luković Z. Partition of genetic trends by origin in Landrace and Large-White pigs. Animal. 2015;9:1605–9. 10.1017/S1751731115001056 26152894

[5] Abdollahi-Arpanahi R, Lourenco D, Legarra A, Misztal I (2021). Dissecting genetic trends to understand breeding practices in livestock: a maternal pig line example. Genet Sel Evol.

[6] Sorensen D, Fernando R, Gianola D (2001). Inferring the trajectory of genetic variance in the course of artificial selection. Genet Res.

[7] Lara LADC, Pocrnic I, Oliveira TDP, Gaynor RC, Gorjanc G (2022). Temporal and genomic analysis of additive genetic variance in breeding programmes. Heredity.

[8] Woolliams JA, Berg P, Dagnachew BS, Meuwissen THE (2015). Genetic contributions and their optimization. J Anim Breed Genet.

[9] Gorjanc G, Hickey JM (2018). AlphaMate: a program for optimizing selection, maintenance of diversity and mate allocation in breeding programs. Bioinformatics.

[10] Gorjanc G, Obšteter J, Oliveira TP. AlphaPart: partition/decomposition of breeding values by paths of information. 2022. https://CRAN.R-project.org/package=AlphaPart.

[11] Henderson CR (1976). A simple method for computing the inverse of a numerator relationship matrix used in prediction of breeding values. Biometrics.

[12] Kennedy BW, Schaeffer LR, Sorensen DA (1988). Genetic properties of animal models. J Dairy Sci.

[13] Quaas RL (1988). Additive genetic model with groups and relationships. J Dairy Sci.

[14] Mrode RA (2005). Linear models for the prediction of animal breeding values.

[15] R Core Team. R: a language and environment for statistical computing. Vienna: R Foundation for Statistical Computing; 2021.

[16] García-Cortés LA, Toro MA (2006). Multibreed analysis by splitting the breeding values. Genet Sel Evol.

[17] Sorensen D, Gianola D (2007). Likelihood, Bayesian, and MCMC methods in quantitative genetics.

[18] Obšteter J, Holl J, Hickey JM, Gorjanc G (2021). AlphaPart-R implementation of the method for partitioning genetic trends. Genet Sel Evol.

[19] Misztal I, Tsuruta S, Lourenco DAL, Masuda Y, Aguilar I, Legarra A, et al. Manual for BLUPF90 family programs. University of Georgia. 2018. http://nce.ads.uga.edu/wiki/doku.php?id=documentation. Accessed 15 Mar 2022.

[20] McCulloch CE, Neuhaus JM (2011). Prediction of random effects in linear and generalized linear models under model misspecification. Biometrics.

[21] Lin LI-K (1989). A concordance correlation coefficient to evaluate reproducibility. Biometrics.

[22] Gaynor RC, Gorjanc G, Hickey JM (2021). AlphaSimR: an R-package for breeding program simulations. G3 (Bethesda).

[23] MacLeod IM, Larkin DM, Lewin HA, Hayes BJ, Goddard ME (2013). Inferring demography from runs of homozygosity in whole-genome sequence, with correction for sequence errors. Mol Biol Evol.

[24] Wickham H, Averick M, Bryan J, Chang W, McGowan L, François R, Grolemund G, Hayes A, Henry L, Hester J, Kuhn M, Pedersen T, Miller E, Bache S, Müller K, Ooms J, Robinson D, Seidel D, Spinu V, Takahashi K, Vaughan D, Wilke C, Woo K, Yutani H (2019). Welcome to the Tidyverse. J Open Source Softw.

[25] Pedersen TL. patchwork: the composer of plots 2020. https://CRAN.R-project.org/package=patchwork. Accessed 15 Mar 2021.

[26] Gorjanc G, Gaynor RC, Hickey JM (2018). Optimal cross selection for long-term genetic gain in two-part programs with rapid recurrent genomic selection. Theor Appl Genet.

[27] Covarrubias-Pazaran G, Gebeyehu Z, Gemenet D, Werner C, Labroo M, Sirak S, Coaldrake P, Rabbi I, Kayondo SI, Parkes E, Kanju E, Mbanjo EGN, Agbona A, Kulakow P, Quinn M, Debaene J (2022). Breeding schemes: what are they, how to formalize them, and how to improve them?. Front Plant Sci.

[28] Goldman IL (2013). Biodiversity in plant breeding. Encyclopedia of biodiversity.

[29] Gorjanc G, Jenko J, Hearne SJ, Hickey JM (2016). Initiating maize pre-breeding programs using genomic selection to harness polygenic variation from landrace populations. BMC Genom.

[30] Hidalgo J, Tsuruta S, Lourenco D, Masuda Y, Huang Y, Gray KA, Misztal I (2020). Changes in genetic parameters for fitness and growth traits in pigs under genomic selection. J Anim Sci.

[31] Sorensen DA, Kennedy BW (1984). Estimation of genetic variances from unselected and selected populations. J Anim Sci.

[32] van der Werf JHJ, de Boer IJM (1990). Estimation of additive genetic variance when base populations are selected1. J Anim Sci.

[33] VanRaden PM (2008). Efficient methods to compute genomic predictions. J Dairy Sci.

[34] Powell JE, Visscher PM, Goddard ME (2010). Reconciling the analysis of IBD and IBS in complex trait studies. Nat Rev Genet.

[35] Wright S (1921). Systems of mating. I. The biometric relations between parent and offspring. Genetics.

