# Supplementary material for: A method for partitioning trends in genetic mean and variance to understand breeding practices
Source: Genet Sel Evol. 2023 Jun 2;55:36. doi: 10.1186/s12711-023-00804-3 (PMC10236722; doi:10.1186/s12711-023-00804-3)
Supplement: Supplementary file 1 — Additional file 1: Figure S1. Definition of the statistical model, priors and posteriors: Directed acyclic graph of the pedigree-based model with nI individuals and nY phenotypic records;" title="Click here to edit">) with explicit representation of Mendelian sampling terms;" title="Click here to edit">) and error term;" title="Click here to edit">), where $\sigma ^2_a$σa2 is the additive genetic variance, $a_{f}$af and $a_{m}$am are the parent’s breeding value, 1 represents a vector of ones, $\mu _i$μi the linear predictor, and $\sigma ^2_e$σe2 the residual variance. Figure S2. Definition of the statistical model, priors and posteriors: representation of gender as the path variable. [file 12711_2023_804_MOESM1_ESM.pdf]

Model definition

Thiago de Paula Oliveira; Jana Obšteter; Ivan Pocrnic; Nicolas Heslot; Gregor Gorjanc
April 12, 2023

Statistical model , priors and posteriors

In the paper, we have defined a standard pedigree-based model for observed data given by

$$\begin{aligned} \mathbf{y}|\mathbf{b},\mathbf{a} &\sim N\left(\mathbf{Xb}+\mathbf{Za},\mathbf{I}\sigma_e^2\right), \\ \mathbf{a} &\sim N\left(\mathbf{0},\mathbf{A}\sigma_a^2\right), \end{aligned} \tag{0.1}$$

where $\mathbf{y}$ is a vector of observed phenotypes, $\mathbf{b}$ is a vector of fixed effects with the design matrix $\mathbf{X}$, $\mathbf{a}$ is a vector of breeding values with the design matrix $\mathbf{Z}$, σ_e^2 is a residual variance, $\mathbf{A}$ is pedigree-based relationship matrix and σ_a^2 is genetic variance in the base population.

To improve the visualization of the fitted model and connect it to the simulated data, we built a directed acyclic graph (DAG). The DAG is illustrated in Figure S1 that shows pedigree and phenotypic records displayed in separate plates as a generalization of the case where animals might have a phenotypic record and dotted lines indicating a possibly missing parent in the pedigree. In the pedigree plate, we have nI individuals represented by founders and non-founders, where the founder's breeding values are `a priori` distributed as $p\left(a_k|\sigma_a^2\right) \sim N\left(0,\sigma_a^2\right)$. The breeding value of non-founders given their parents are then represented by $a_k = \frac{1}{2}a_{f(k)} + \frac{1}{2}a_{m(k)} + w_k$, where $a_{f(k)}$ and $a_{m(k)}$ are parent's breeding values and w_k is the Mendelian sampling term $p\left(w_k|\mathbf{W}_{k,k}\right) \sim N\left(0,\mathbf{W}_{k,k}\sigma_a^2\right)$. Moreover, we have considered only intercept as the fixed effect in the linear predictor.

Following the Figure S1, we show that matrix $\mathbf{A}$ can be decomposed as $\mathbf{A} = \mathbf{TWT}^T$ using the LDL decomposition [11,12,13,14]. The diagonal elements of $\mathbf{W}$ can be computed according to specific scenarios described by [11,12,13,14] as i) $\mathbf{W}_{k,k} = \frac{1}{2} - \frac{1}{4}\left(F_{f(k)} + F_{m(k)}\right)$ when both

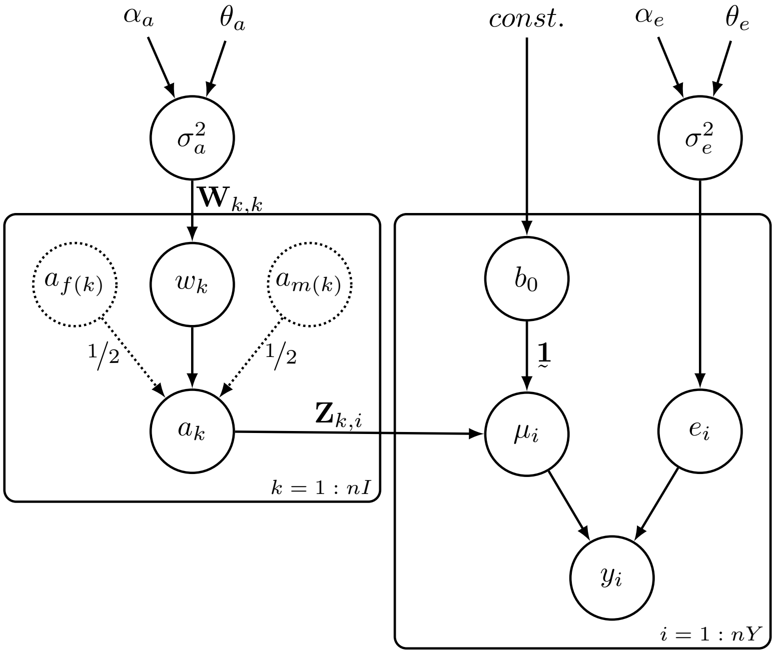

Figure S1: Directed acyclic graph of the pedigree-based model with nI individuals and nY phenotypic records (y_i) with explicit representation of Mendelian sampling terms (w_k) and error term (e_i), where σ_a^2 is the additive genetic variance, $a_{f(k)}$ and $a_{m(k)}$ are parent's breeding value, **1** represents a vector of ones, μ_i the linear predictor, and σ_e^2 the residual variance

parents are known; ii) $\mathbf{W}_{k,k} = \frac{3}{4} - \frac{1}{4}F_{m(k)}$ or $\mathbf{W}_{k,k} = \frac{3}{4} - \frac{1}{4}F_{f(k)}$ when one parent is known; and iii) $\mathbf{W}_{k,k} = 1$ when both parents are unknown, where $F_{f(k)}$ and $F_{m(k)}$ are respectively the coefficients of inbreeding of the father and mother of the individual k [11,12,13,14]. While this theory is standard, some quantitative genetics software still ignore parental inbreeding coefficients in setting up the $\mathbf{A}^{-1}$, which can have significant impact on the analysis of genetic variance as we will show in results.

We used the full Bayesian approach to infer breeding values using equation (1.1). To this end, we specified prior distribution for all model parameters, as shown in Figure S1. Thus, $\mathbf{b}$, σ_a^2, and σ_e^2 were assumed to have a prior joint density of the form $p\left(\mathbf{b}, \sigma_a^2, \sigma_e^2\right) = p\left(\mathbf{b}\right) p\left(\sigma_a^2\right) p\left(\sigma_e^2\right)$ with a flat distribution for b and a conjugate inverse-gamma(α, θ) distribution for variances (or gamma distribution for precisions = 1/variance), where α and θ are set to a value such as

0.1^3:

$$p(\mathbf{b}) \propto const.,$$
$$p\left(\tau_a = 1/\sigma_a^2 | \alpha_a, \theta_a\right) \propto \tau_a^{\alpha_a - 1} \exp\left(-\theta_a \tau_a\right),$$
$$p\left(\tau_e = 1/\sigma_e^2 | \alpha_e, \theta_e\right) \propto \tau_e^{\alpha_e - 1} \exp\left(-\theta_e \tau_e\right),$$

with $\tau_a > 0$, $\alpha_a \geq 0$, $\theta_a \geq 0$, $\tau_e > 0$, $\alpha_e \geq 0$ and $\theta_e \geq 0$. The posterior distribution is then obtained by applying Bayes' theorem conditional on the data:

$$p\left(\mathbf{b}, \mathbf{a}, \sigma_a^2, \sigma_e^2 | \mathbf{y}\right) \propto p\left(\mathbf{y} | \mathbf{b}, \mathbf{a}, \sigma_e^2\right) p(\mathbf{b})\, p\left(\mathbf{a} | \mathbf{A}, \sigma_a^2\right) \times$$
$$p\left(\sigma_a^2 | \alpha_a, \theta_a\right) p\left(\sigma_e^2 | \alpha_e, \theta_e\right).$$

DAG as an insightful way to represent paths

The pedigree plate in the DAG representation in Figure S1 also shows the formations of paths that can be explored using the partitioning methodology. It is, in fact, highly related to Wright's path coefficients described in [35]. To illustrate this idea, let a_k be a female animal. Thus the Mendelian sampling term w_k gets assigned to the animal's path, in this case, the female gender as shown in Figure S2. Consequently, the female path for animal a_k is represented by

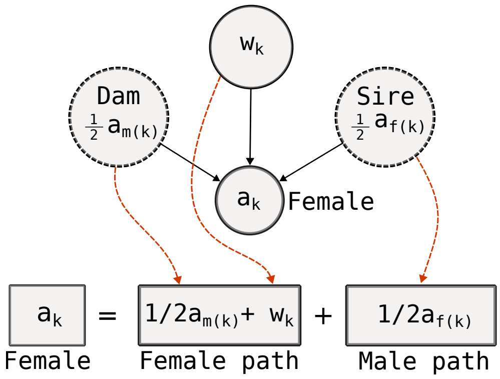

Figure S2: Representation of gender as the path variable

$1/2a_{m(k)} + w_k$ while male path by $1/2a_{f(k)}$.
