# Supplementary figures and images for: A method for partitioning trends in genetic mean and variance to understand breeding practices
Source: Genet Sel Evol. 2023 Jun 2;55:36. doi: 10.1186/s12711-023-00804-3 (PMC10236722; doi:10.1186/s12711-023-00804-3)

# Medium accuracy

Path: ■ F ■ M – Non-Selected ■ M – Selected ■ Sum

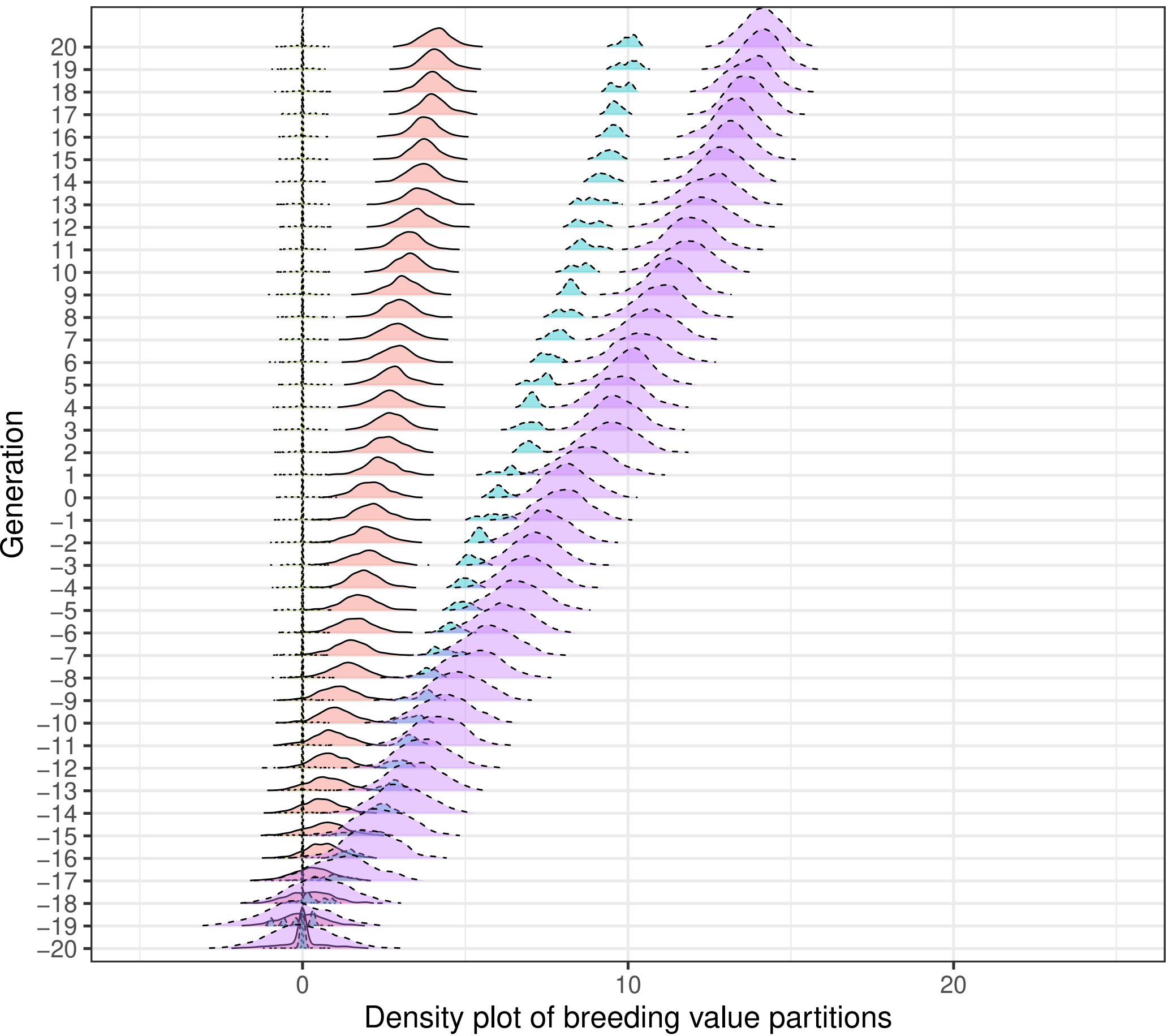

Supplement: Supplementary file 2 — Additional file 2: Figure S3. Distribution of breeding value partitions by sex and selection status [selected males), non-selected males), and females] over generations for medium-accuracy scenario [35]. [file 12711_2023_804_MOESM2_ESM.pdf]

# High accuracy

Path: ■ F ■ M – Non-Selected ■ M – Selected ■ Sum

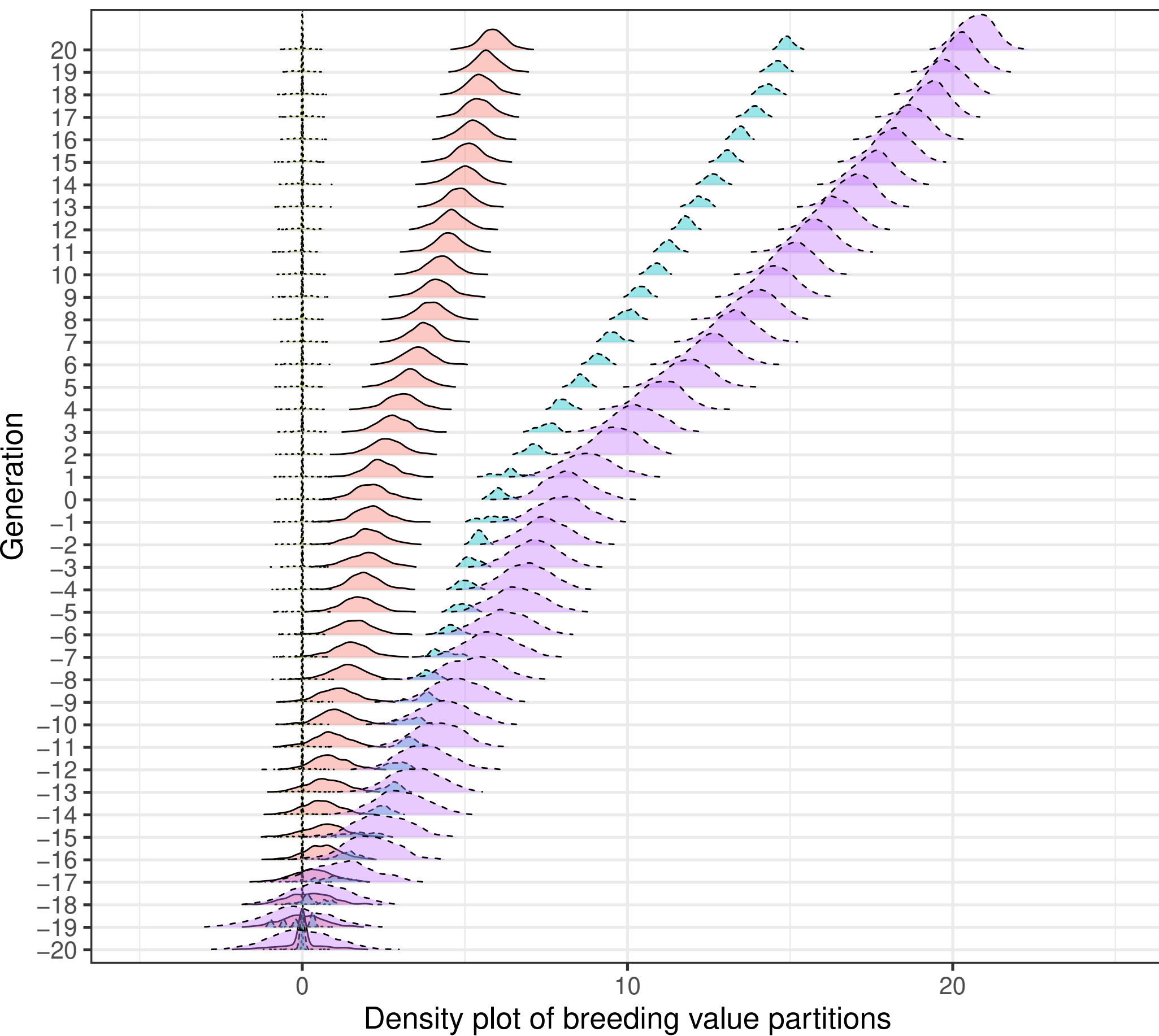

Supplement: Supplementary file 3 — Additional file 3: Figure S4. Distribution of breeding value partitions by sex and selection status [selected males), non-selected males), and females] over generations for high-accuracy scenario. [file 12711_2023_804_MOESM3_ESM.pdf]

Correlation between the F and M(S) breeding values partition

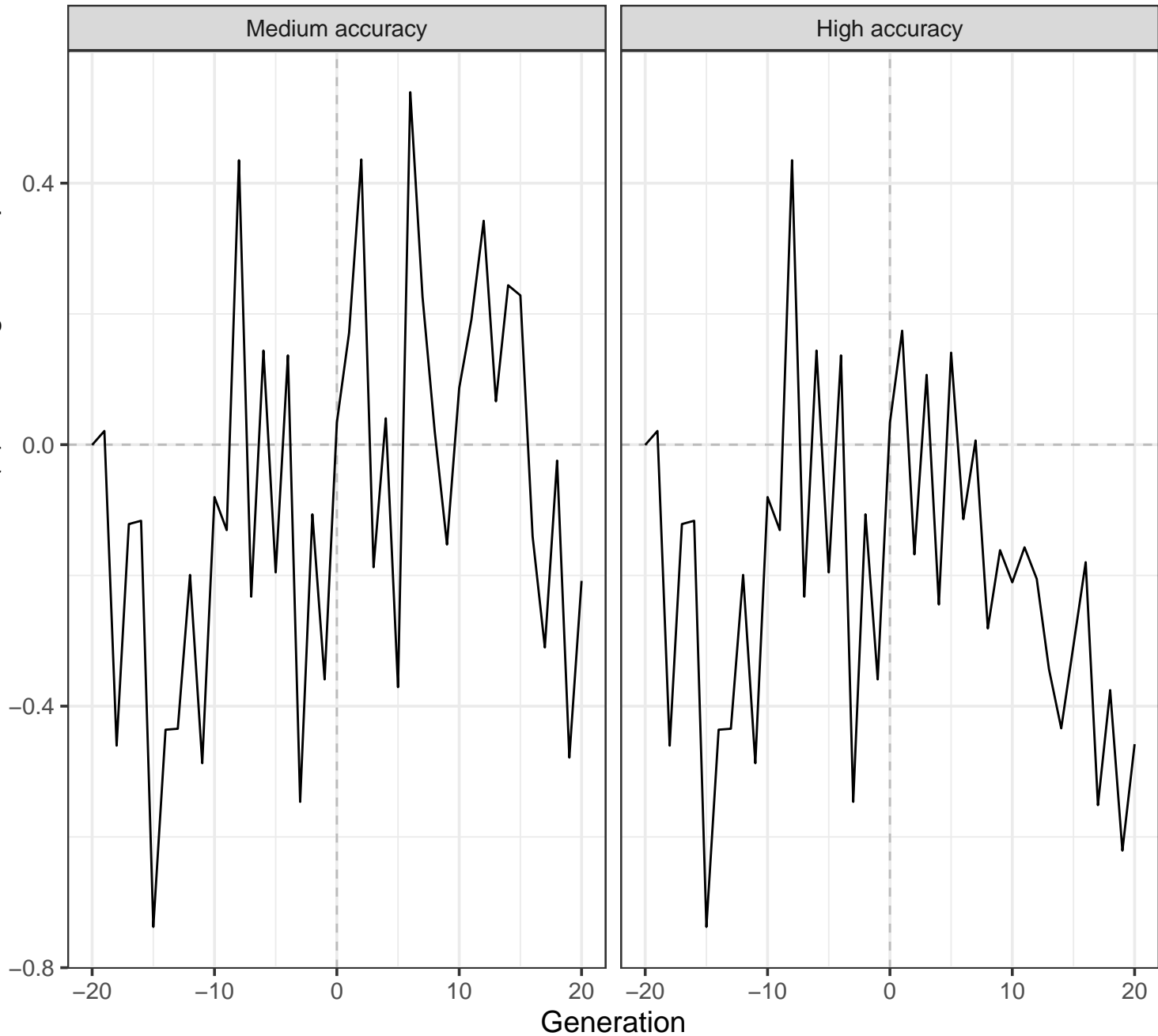

Supplement: Supplementary file 4 — Additional file 4: Figure S5. Correlation between females (F) and selected males (M(S)) partitions using true breeding values for the medium- and high-accuracy scenarios and one simulation replicate. [file 12711_2023_804_MOESM4_ESM.pdf]

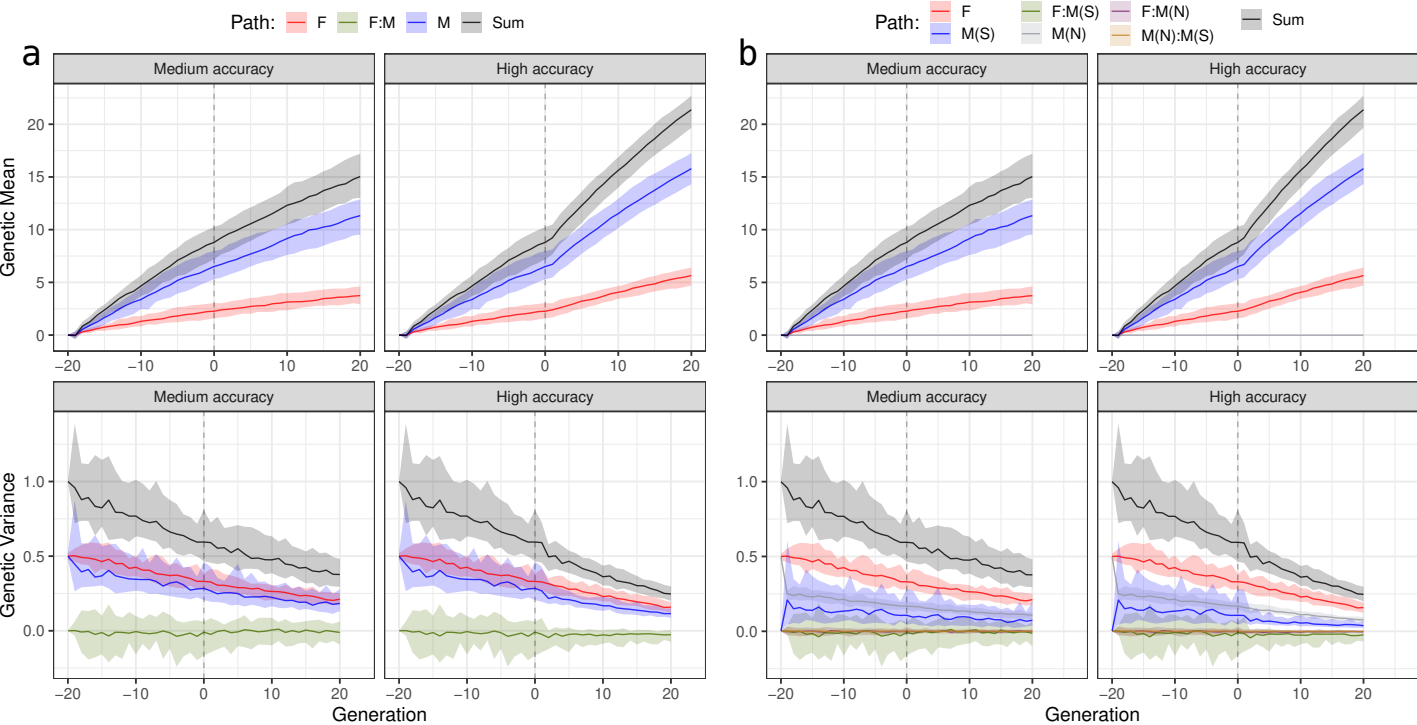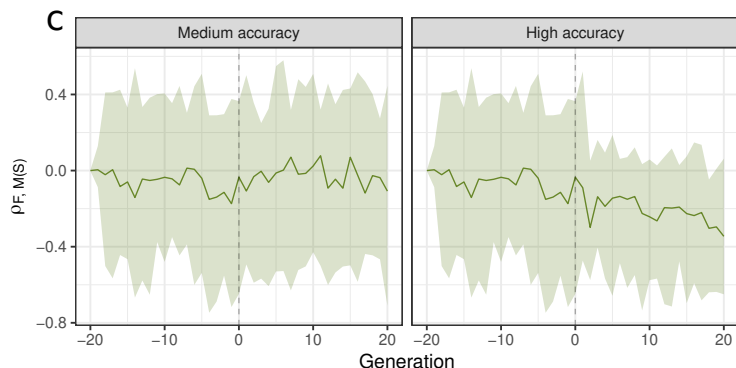

Supplement: Supplementary file 5 — Additional file 5: Figure S6. Partitions of genetic mean and variance bysex,by sex and selection status [selected males), non-selected males), and females], andthe Pearson correlation between F and Mpartitions for the medium- and high-accuracy scenarios by sex and selection status using true breeding values for 30 simulation replicates. [file 12711_2023_804_MOESM5_ESM.pdf]

# Medium Accuracy

Generation: 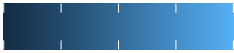 -20 -10 0 10 20

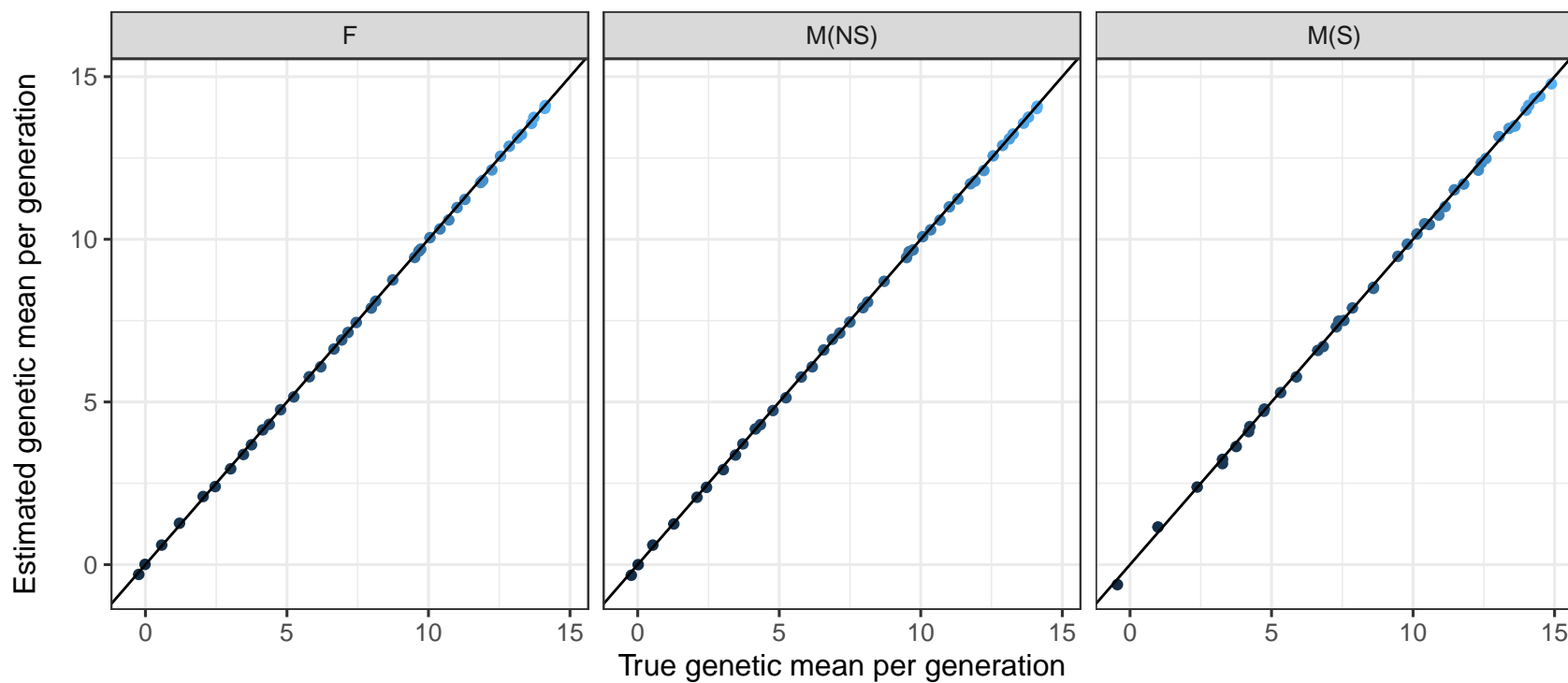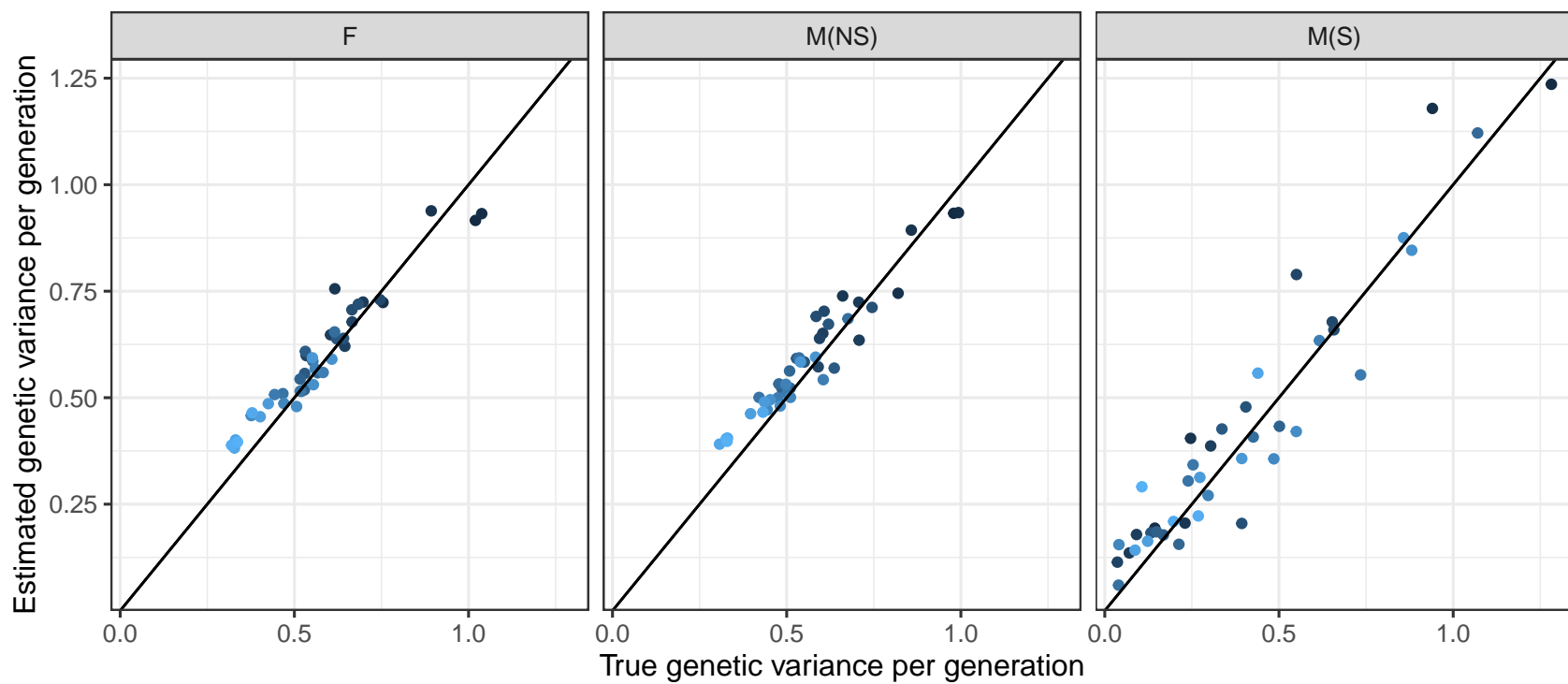

Supplement: Supplementary file 6 — Additional file 6: Figure S7. Estimated and true genetic means and variances over 40 generations by selected males), non-selected males), and femalesin the medium-accuracy scenario. The solid line represents the equality line $y=x$y=x, and the dots are the Cartesian coordinates of estimated and true values. [file 12711_2023_804_MOESM6_ESM.pdf]

High accuracy

Generation: 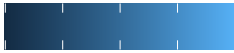 -20 -10 0 10 20

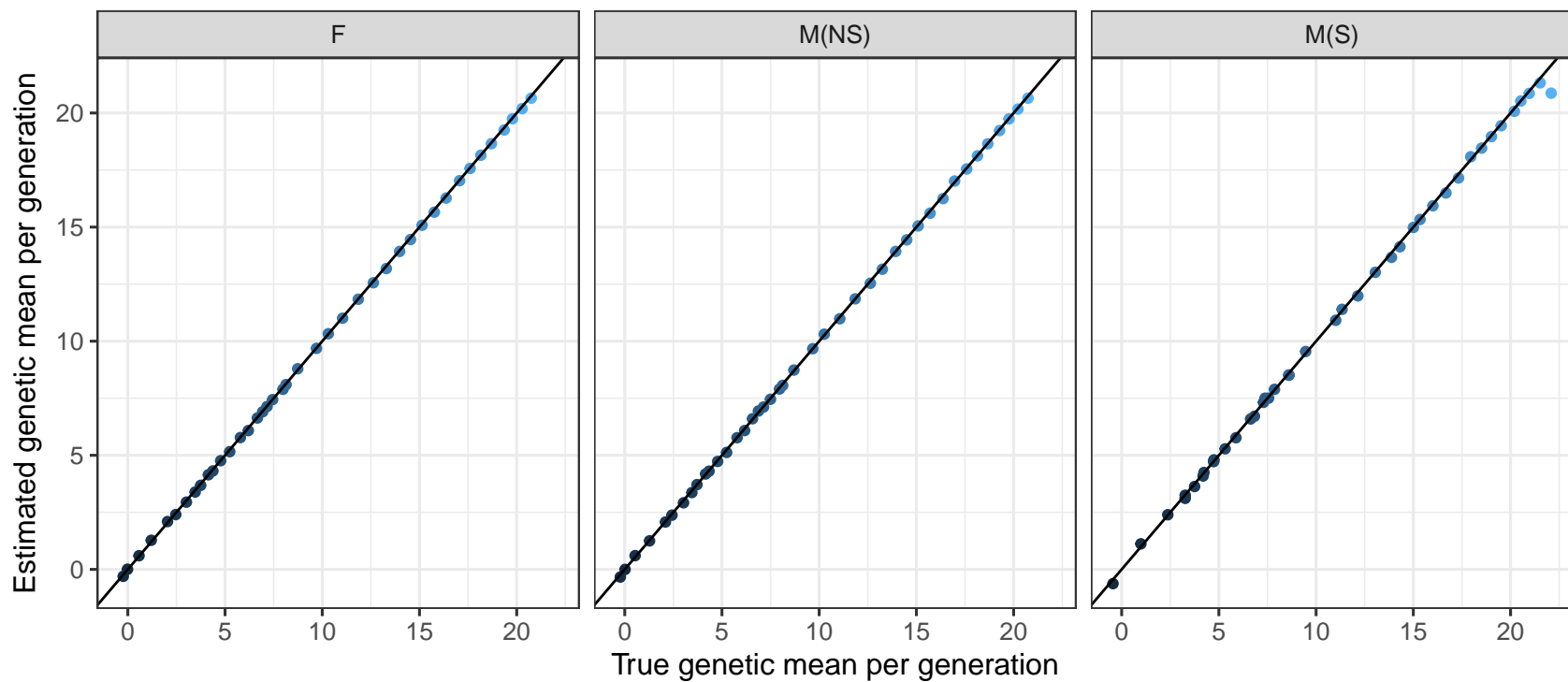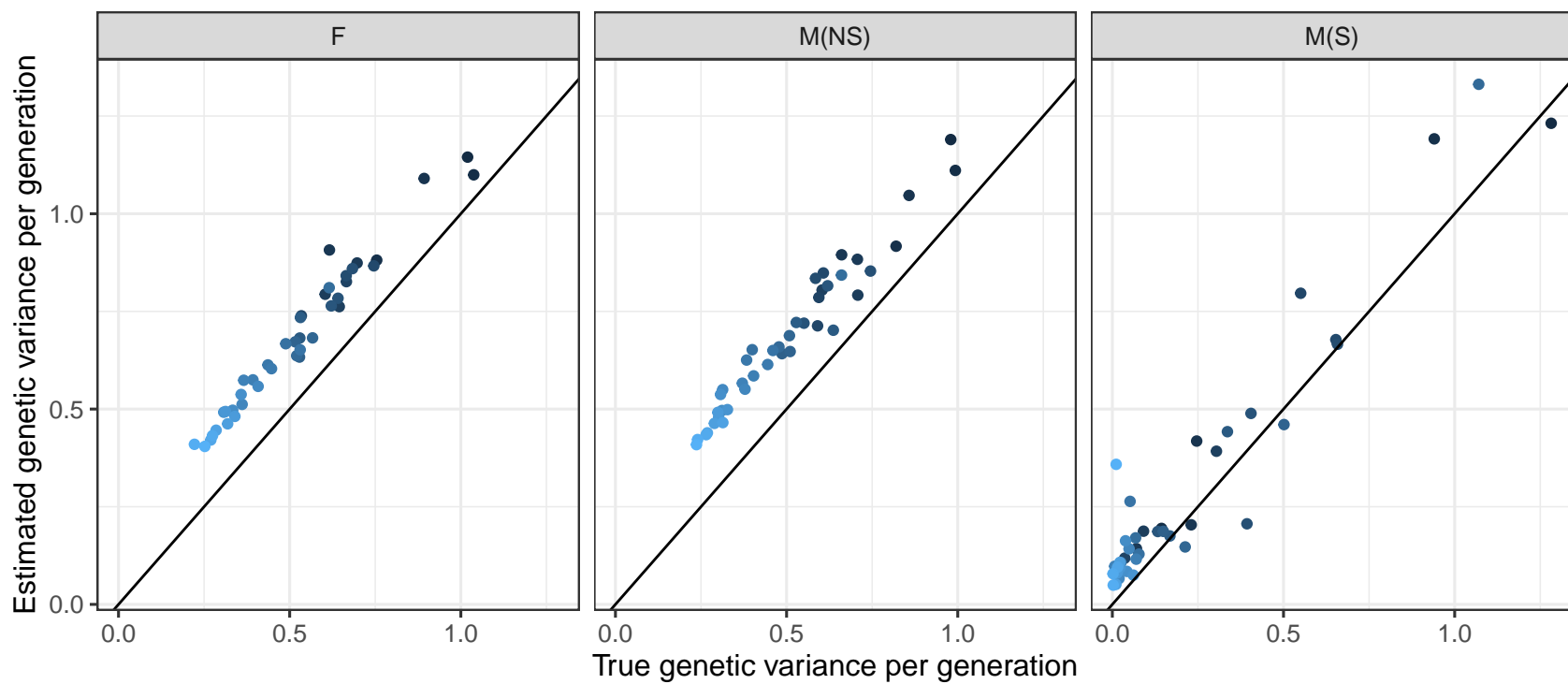

Supplement: Supplementary file 7 — Additional file 7: Figure S8. Estimated and true genetic means and variances over 40 generations by selected males), non-selected males), and femalesin the high-accuracy scenario. The solid line represents the equality line $y=x$y=x, and the dots are the Cartesian coordinates of estimated and true values. [file 12711_2023_804_MOESM7_ESM.pdf]

Medium Accuracy

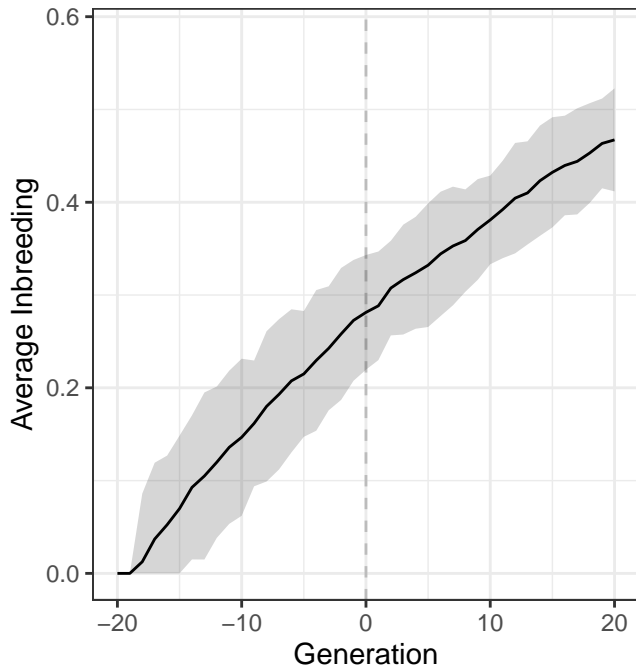

High Accuracy

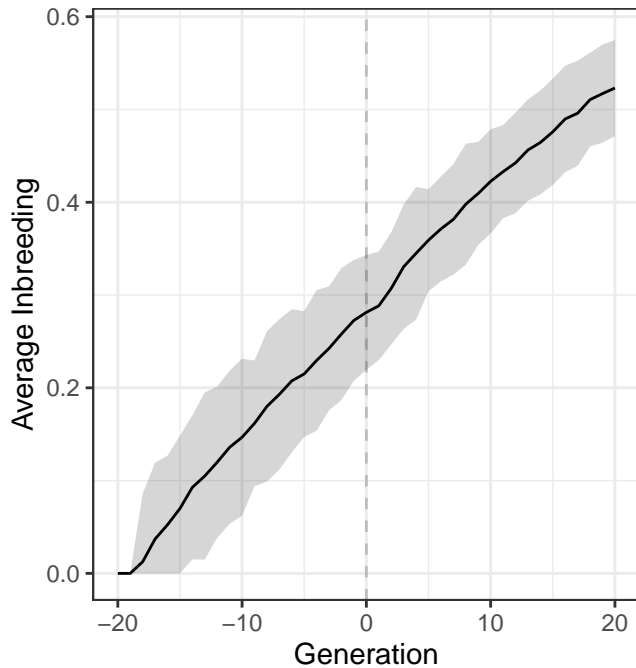

Supplement: Supplementary file 8 — Additional file 8: Figure S9. Pointand intervalestimates for inbreeding over generation considering all animals in a specific generation. [file 12711_2023_804_MOESM8_ESM.pdf]

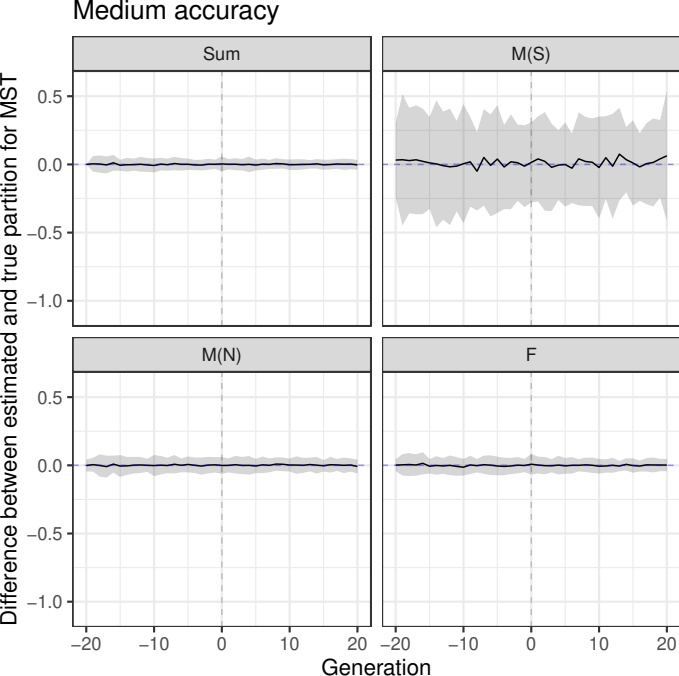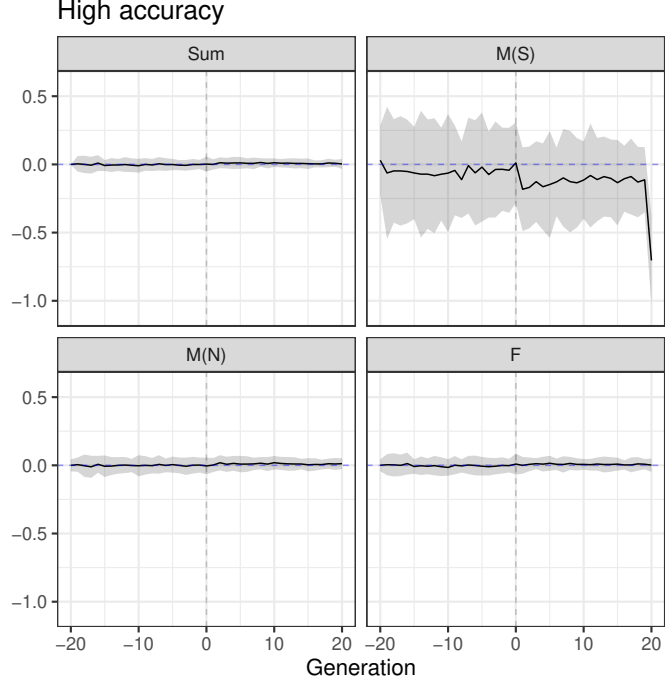

Supplement: Supplementary file 9 — Additional file 9: Figure S10. The difference between true and estimated Mendelian sampling termsis distributed over generations. The totalis partitioned by selected males), non-selected males), and femalespaths in the medium- and high-accuracy selection scenario. We are considering 30 replicates (zero value is denoted with a dashed line and mean differenceover replicates is denoted with a solid line, and 95% quantile of differences over replicates is denoted with a ribbon). [file 12711_2023_804_MOESM9_ESM.pdf]

Medium accuracy

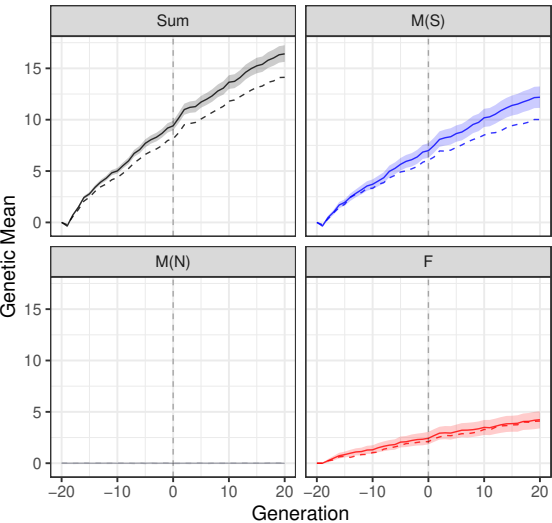

High accuracy

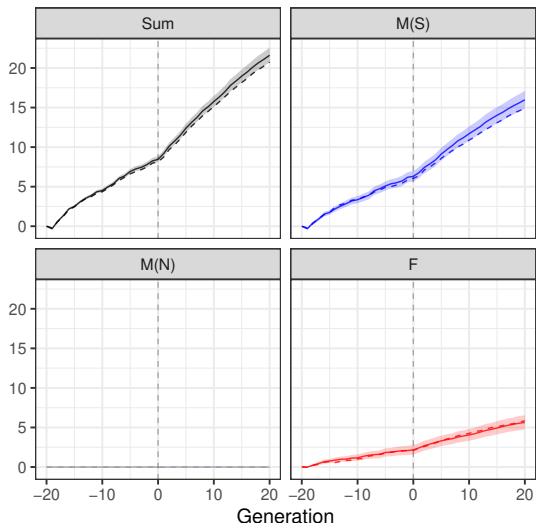

Supplement: Supplementary file 10 — Additional file 10: Figure S11 Partitioning of the total genetic meanover generations by selected males), non-selected males), and femalespaths in the medium-accuracy and high-accuracy scenario. We considered one replicate without accounting for inbreeding in the model (true value is denoted with a dashed line and posterior mean denoted with a solid line, and 95%credible interval is denoted with a ribbon). [file 12711_2023_804_MOESM10_ESM.pdf]

Contribution of Mendelian Sampling Term to Genetic Mean

Medium accuracy

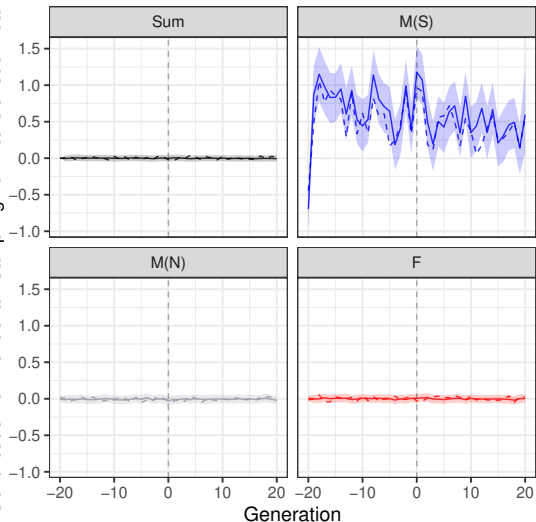

High accuracy

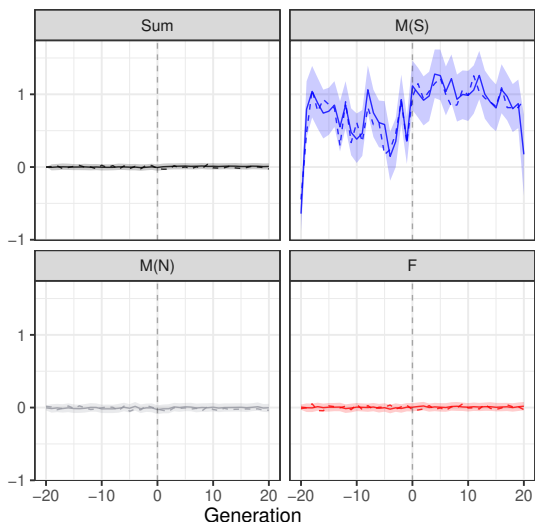

Supplement: Supplementary file 11 — Additional file 11: Figure S12. Partitioning of the total Mendelian Sampling termover generations by selected males), non-selected males), and femalespaths in the medium-accuracy and high-accuracy scenario. We considered one replicate without accounting for inbreeding in the model (true value is denoted with a dashed line and posterior mean denoted with a solid line,and 95% credible interval is denoted with a ribbon). [file 12711_2023_804_MOESM11_ESM.pdf]

Medium accuracy

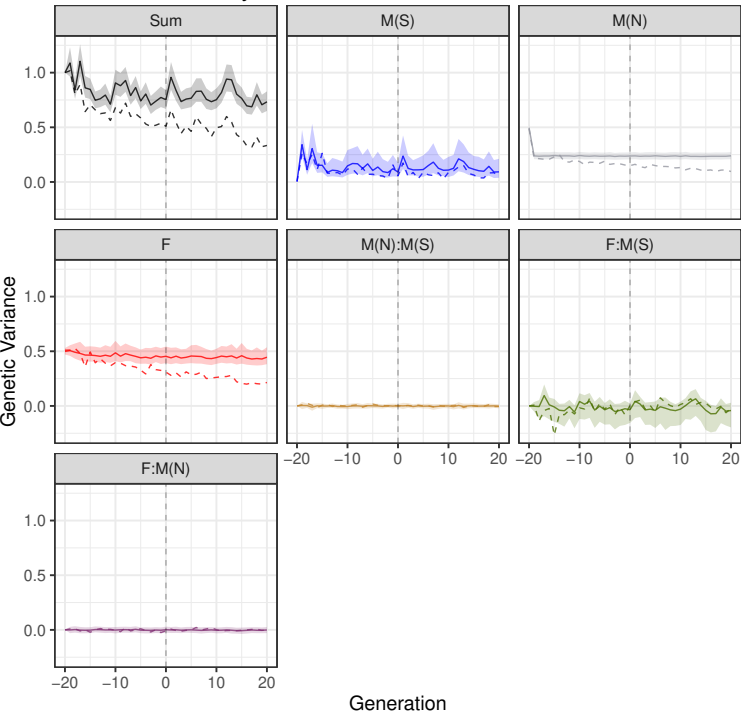

High accuracy

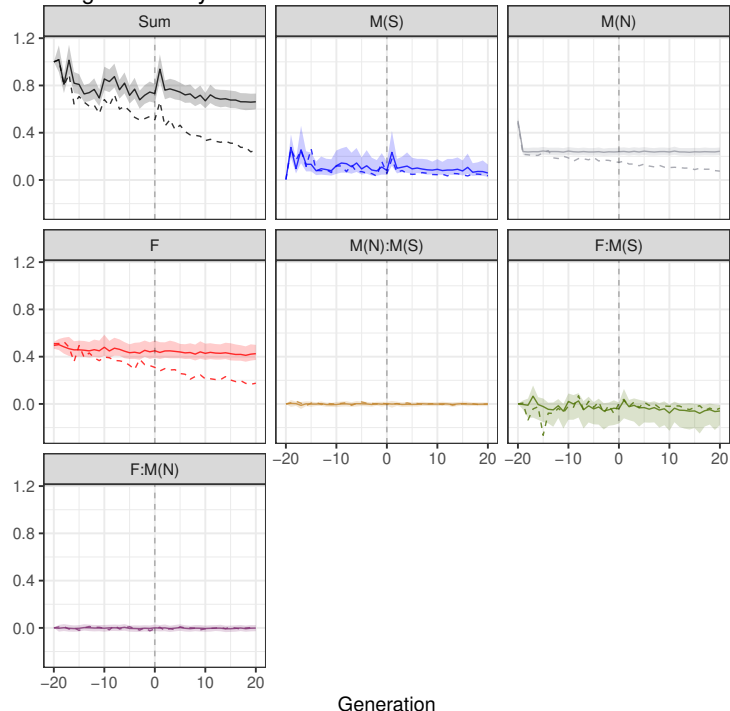

Supplement: Supplementary file 12 — Additional file 12: Figure S13. Partitioning of the total genetic varianceover a generation by selected males), non-selected males), and femalespath in the medium-accuracy and high-accuracy scenario. We considered one replicate without accounting for inbreeding in the model (true value is denoted with a dashed line and posterior mean denoted with a solid line, and 95%credible interval is denoted with a ribbon). [file 12711_2023_804_MOESM12_ESM.pdf]

- Individual 1
- Individual 2
- Individual 3

Path:  Domestic  Imported

a

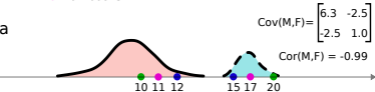

b

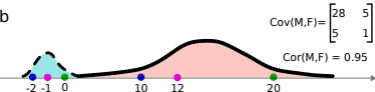

Supplement: Supplementary file 13 — Additional file 13: Figure S14. Example ofnegative andpositive covariance partitions. [file 12711_2023_804_MOESM13_ESM.pdf]
